# Overactivation of EGFR signaling in skeletal stem/progenitor cells promotes bone formation and repair

**DOI:** 10.7150/thno.115406

**Published:** 2025-07-24

**Authors:** Yuxiang Hu, Yangyang Chen, Xiaoyao Peng, Haitao Li, Guosilang Zuo, Hao Xu, Fashuai Wu, Yi Wang, Zengwu Shao, Yulong Wei

**Affiliations:** 1 Department of Orthopedics, Union Hospital, Tongji Medical College, Huazhong University of Science and Technology, Wuhan, 430022, Hubei, China.; 2 Department of Orthopedics, The First Affiliated Hospital of Zhengzhou University, Zhengzhou, 450052, China.; 3 Department of Orthopedics, Affiliated Hospital of Guizhou Medical University, Guiyang, 55000, China.; 4 Department of Radiology, Zhongnan Hospital of Wuhan University, Wuhan, 430071, Hubei, China.

**Keywords:** bone regeneration, EGFR signaling, HBEGF, Prx1, periosteal progenitors

## Abstract

**Background:** Epidermal growth factor receptor (EGFR) signaling plays an important role in bone development. However, knowledge of its specific function in skeletal stem cells during bone healing remains scant.

**Methods:** We used a lineage tracing approach and a stem/progenitor cell-specific EGFR overactivation mouse model which is generated by overexpressing heparin-binding EGF-like growth factor (HBEGF), an EGFR ligand, in Prx1-cre mice (*HBEGF Over^Prx1^*), to analyze the crucial roles of EGFR signaling in periosteal progenitor cells during fracture healing.

**Results:** Compared with wild type, *HBEGF Over^Prx1^* mice are found to have thicker trabecular and cortical bone structure and exhibit accelerated fracture healing. Single-cell RNA sequencing reveals that *HBEGF* is highly expressed in a periosteal progenitor cluster that constitutes a large portion of the callus cells and lays at the center of a developmental path that gives rise to chondrocytes and osteoblasts within the callus. *In vitro* experiments further demonstrate that periosteal progenitors isolated from *HBEGF Over^Prx1^* mice display strong chondrogenic, osteogenic and angiogenic abilities, thus promoting fracture healing. Treating mice with gefitinib, an EGFR inhibitor, completely abolishes the promotional effects in *HBEGF Over^Prx1^* mice.

**Conclusion:** Our data reveal a cellular mechanism of EGFR signaling underlying fracture healing, and suggest that targeting EGFR may provide a potential therapeutic tool for delayed fracture healing or fracture non-union.

## Introduction

Insufficient bone repair and regeneration are very common in patients with major trauma or severe illness and often lead to pain, arthrosis, and disability 1. Approximately 5.6 million fractures occur per year in the United States, and complications of healing such as nonunion, malunion, osteomyelitis, and chronic pain occur in 5-10% of these 2, resulting in a substantial physical, medical, emotional, and financial burden upon affected individuals and our society as a whole 3, 4. Despite new advances in orthopedic surgery which have substantially improved fracture healing outcomes, there is still a subset of fractures which fail to heal as expected 5, 6. Therefore, understanding the molecular and cellular mechanisms during fracture healing may identify new therapeutic targets to promote bone repair.

Complex coordination among inflammatory cells, stem cells, osteoblasts, chondrocytes, osteoclasts, and endothelial cells together with surrounding cytokines and growth factors is involved in the cellular and molecular events during fracture healing 7. Fracture healing begins with a robust inflammatory response followed by formation of a hematoma, which secretes vital cytokines and growth factors to attract the progenitor cells to the fracture site, where they proliferate and differentiate into cartilage-forming chondrocytes and bone-forming osteoblasts 8. Many sources of progenitor cells have been reported to directly participate in bone repair, including bone marrow, endosteal, muscle and adipose tissues surrounding fractured bone as well as blood vessel walls. However, these cells recruited systemically are minimal contributors to cartilage and bone, but give rise mostly to inflammatory cells and osteoclasts 9, 10. The periosteum is the tissue covering most of the outer surface of the bone. It is physically separated from the bone marrow compartment and appears to have a much greater regenerative capacity, playing a critical role in fracture healing 11. Many markers have been utilized to identify progenitor cells from periosteum including paired-related homeobox gene-1 (Prx1) 12, alpha smooth muscle actin (αSMA) 13 and glioma-associated oncogene homolog 1 (Gli1) 14. Among them, Prx1 is a homeobox gene expressed in several developing tissues, especially skeletal elements 15. Recent studies reported that Prx1 has been utilized to identify mouse osteochondral progenitor cells, and are localized within periosteum, bone marrow and skeletal muscle, plays an essential role in bone development and repair 16-19. Although Prx1 as a marker of periosteal progenitor cells has been well identified, most studies have focused on tracing Prx1-derived cells (or their progeny) during fracture repair 20. The functional role of Prx1 and the contribution of periosteal progenitor cells expressing Prx1 to the fracture repair process have so far not been fully elucidated.

If the identification of periosteal progenitors is critical, equally important are the growth factors that regulate periosteal progenitors during fracture repair. Notably, the Epidermal growth factor (EGF) family ligands, including EGF, TGF-α, heparin-binding EGF (HBEGF), betacellulin (BTC), and epiregulin, act on the EGF receptor (EGFR) to regulate a variety of cell behaviors, such as proliferation, survival, migration, and differentiation 21, 22. EGFR signaling plays a critical role in skeletal development, which involves coordinated bone formation and resorption. Mice with knockdown of EGFR activity in progenitors and osteoblasts, or treated with an EGFR-specific inhibitor, experience delayed formation of the secondary ossification center and recruitment of osteoblasts and osteoclasts, leading to osteoporosis 23, 24. Meanwhile, overexpression of EGFR leads to a high cortical bone mass phenotype 25. Although the EGFR-mediated signaling pathway has been proven to be vital in bone metabolism, its function in Prx1^+^ skeletal stem/progenitor cells and the downstream mechanism during bone repair have been poorly delineated.

Here, we first used Prx1 to identify mouse skeletal stem/progenitor cells. Next, we genetically enhanced EGFR activity by adopting a Rosa-diphtheria toxin receptor (DTR) model that is normally used for cell ablation 26. DTR was originally used as a receptor for bacterial diphtheria toxin, later found to be human full-length heparin-binding EGF like growth factor (HBEGF), a ligand for EGFR, which shares 81% sequence identity with the mouse ortholog 27, 28. We crossed Rosa-DTR mice with stem/progenitor cell-specific Prx1-Cre mice to generate Prx1-HBEGF mice. Cre-mediated excision of the floxed stop codon caused HBEGF expression in Prx1^+^ cells and their progenies under the control of the ROSA26 promoter 29, allowing us to investigate the effect of stem/progenitor cell-specific EGFR overexpression during bone regeneration and repair.

## Results

### EGFR overactivation in stem/progenitor cells promotes bone formation

**Figure 1:**
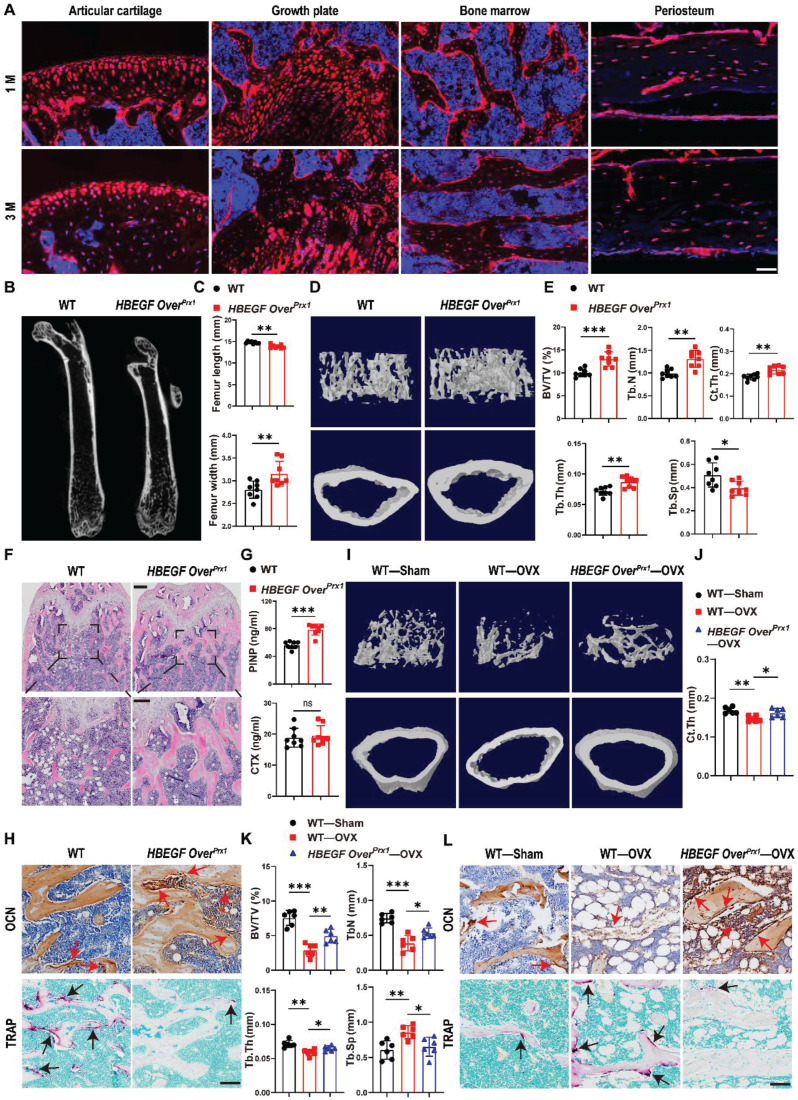
** Activation of EGFR signaling contributes to higher trabecular and cortical bone contents.** (A) Tracing of Prx1 lineage cells on femoral frozen sections of 1- and 3-month-old Prx1-Cre; tdTomato mice. Red: tdTomato^+^ cells; blue: nuclear staining by DAPI. Scale bars = 50 µm. (B) Micro-CT images of femurs from 3-month-old WT and *HBEGF Over^Prx1^* mice. (C) Quantification of femur length and width of WT and *HBEGF Over^Prx1^* mice at 3 months old; n = 8 per group. (D) Representative 3D reconstructed micro-CT images of trabecular architecture and cortical bone from 3-month-old WT and *HBEGF Over^Prx1^* distal femurs. (E) Quantification of the bone parameters including trabecular bone volume fraction (BV/TV), trabecular number (TB. N), trabecular thickness (TB.TH), trabecular separation (TB. SP) and cortical thickness (CT. TH) based on micro-CT; n = 8 per group. (F) Representative H&E staining images of distal femurs from 3-month-old WT and* HBEGF Over^Prx1^* mice. Magnified images of the boxed areas are shown in the panel below. Scale bar = 500 μm (upper image); 100 μm (lower image). (G) Serum PINP and CTX levels in WT and *HBEGF Over^Prx1^* mice analyzed by ELISA; n = 8 per group. (CTX, *P* = 0.6441). (H) Immunohistochemical staining of OCN and TRAP staining of trabecular bone sections from distal femurs of WT and *HBEGF Over^Prx1^* mice. OCN-positive cells or TRAP-positive cells in the distal femurs are indicated by red or black arrows, respectively; scale bar = 50 µm. (I) Representative 3D reconstructed micro-CT images of trabecular architecture and cortical bone at 4 weeks post OVX surgery from WT and *HBEGF Over^Prx1^* distal femurs. (J) Quantification of cortical thickness (CT. TH) based on micro-CT; n = 6 per group. (K) Quantification of the bone parameters including BV/TV, TB. N, TB.TH and TB. SP based on micro-CT; n = 6 per group. (L) Immunohistochemical staining of OCN and TRAP staining on trabecular bone sections at 4 weeks post OVX surgery from distal femurs of WT and *HBEGF Over^Prx1^* mice. OCN-positive cells or TRAP-positive cells in the distal femurs are indicated by red or black arrows; scale bar = 50 µm. Data are presented as means ± SD. Statistical analysis was performed using two-tailed Student's t test (C, E, G) and one-way ANOVA with Bonferroni's post-hoc test for multiple comparisons (J, K). ns = not significant, *P < 0.05, **P < 0.01, ***P < 0.001.

To determine whether postnatal Prx1^+^ cells contribute to the formation of chondrocytes and osteoblasts, we first generated Prx1-Cre; Rosa-tdTomato mice to trace Prx1 lineage in 1- and 3-month-old mice. Prx1-labeled cells were abundant throughout the articular cartilage, the height of the growth plate, and the cortical bone, especially the periosteal surface, as well as the cancellous bone (Figure 1A), suggesting that Prx1 marks a major skeletal progenitor pool that gives rise to chondrocytes and osteoblasts. Next, to target EGFR signaling in stem/progenitor cells during bone development and remodeling, we bred Prx1-cre and Rosa-DTR mice to generate a Prx1-HBEGF overexpressing mouse line (*HBEGF Over^Prx1^*). Western blots confirmed increased HBEGF expression as well as elevated phospho-EGFR (p-EGFR) in *HBEGF Over^Prx1^* mouse bone tissues (Figure S1A). At 3 months of age, these mice showed similar body weight in comparison with their wild-type (WT) siblings (Figure S1B). Notably, the long bones in *HBEGF Over^Prx1^* mice were shorter and thicker (Figure 1B-C). Micro-CT analysis of distal femurs revealed that *HBEGF Over^Prx1^* mice showed a significant increase in trabecular bone mass and cortical bone thickness at 3 months of age, compared to WT mice (Figure 1D). Bone structural parameters based on micro-CT, including bone volume fraction (BV/TV), trabecular number (TB. N), trabecular thickness (TB. TH) and cortical thickness (Ct. TH) were all markedly increased by 30%, 33%, 21% and 17%, respectively, whereas trabecular separation (TB. SP) was accordingly decreased in* HBEGF Over^Prx1^* mice (23%) compared to WT mice (Figure 1E).

Similarly, H&E staining of the femurs further confirmed a marked high-bone-mass phenotype with increased trabecular bone volume in *HBEGF Over^Prx1^ mice* (Figure 1F). To understand the mechanism underlying the trabecular bone changes, we assayed typical serum markers of bone formation-pro-collagen type I N-terminal propeptide (PINP) and osteocalcin (OCN), and of bone resorption-tartrate-resistant acid phosphatase (TRAP) and C-terminal telopeptide of type I collagen (CTX). Compared with WT mice, the serum levels of PINP and OCN were dramatically increased, whereas TRAP and CTX levels remained unaffected in *HBEGF Over^Prx1^* mice (Figure 1G and S1C). Moreover, we performed immunostaining of OCN as well as TRAP staining of the bone sections. Results showed that the numbers of OCN-positive cells on the bone surface and in the bone marrow were both dramatically increased in *HBEGF Over^Prx1^* mice (Figure 1H and S1D), while the numbers of TRAP-positive osteoclasts were significantly lower than WT mice (Figure 1H and S1D). These results indicated that the combination of the increase in bone formation and the decrease in bone resorption resulted in the high-bone-mass phenotype in *HBEGF Over^Prx1^* mice.

Postmenopausal osteoporosis is associated with decreased osteoblastic activity and increased osteoclastic bone resorption 30. We next investigate the role of EGFR overactivation in osteoporosis-induced bone loss. Ovariectomy (OVX) was performed on 2-month-old female WT and *HBEGF Over^Prx1^* mice to imitate postmenopausal osteoporosis. Micro-CT and H&E analysis revealed that, compared to the sham group, bone mass loss was significantly higher in the OVX group mice compared to the sham group, but significantly reduced in the *HBEGF Over^Prx1^* OVX mice (Figure 1I and S2A-C). Quantitative analyses of BV/TV, TB. TH, TB. N, Ct. TH and TB. SP based on micro-CT further confirmed the preventative efficacy of EGFR overactivation on OVX-induced bone loss (Figure 1J-K and S2A). Immunohistochemical staining of OCN and TRAP staining showed that osteogenic activity was slightly reduced, whereas osteoclast activity was accordingly increased in OVX mice (Figure 1L and S2D). However, these effects were all significantly attenuated in *HBEGF Over^Prx1^* OVX mice, suggesting that EGFR overactivation could protect against osteoporosis-induced bone loss.

### EGFR overactivation in stem/progenitor cells accelerates fracture healing

**Figure 2:**
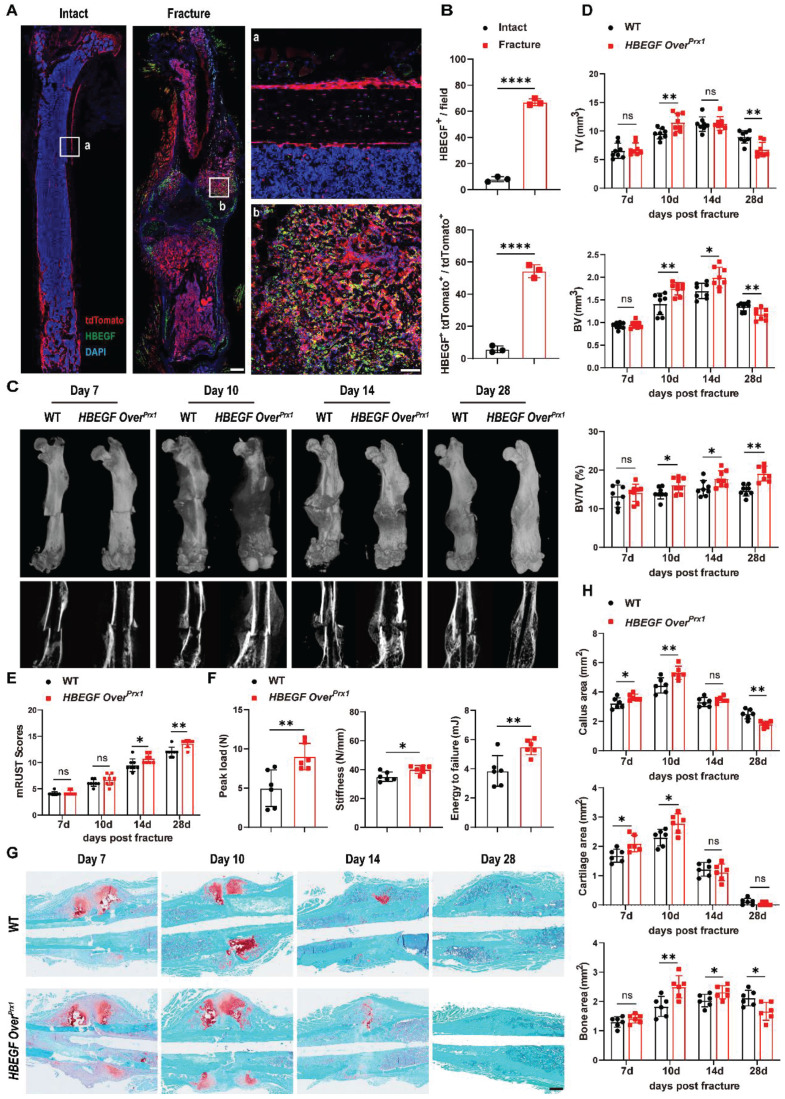
** Activation of EGFR signaling accelerates fracture healing.** (A) Immunofluorescence images of HBEGF distribution in intact and fractured Prx1-Cre; tdTomato mouse femurs. Boxed areas in the left panel are shown at higher magnification on the right. Red: tdTomato^+^ cells; green: HBEGF^+^ cells; blue: nuclear staining by DAPI. Scale bars = 500 µm or 50 µm. (B) Percentage of HBEGF^+^ and tdTomato^+^HBEGF^+^ over tdTomato^+^ within callus were calculated; n = 3 per group. (C) Representative 3D reconstructions and coronal cross-sectional micro-CT images of fracture callus at 7, 10, 14 and 28 dpf. (D) The tissue volume (TV), bone volume (BV) and bone volume fraction (BV/TV) of fracture callus at 7, 10, 14 and 28 dpf were analyzed; n = 8 per group. (E) Fracture healing scores were quantified based on mRUST scoring criteria at 7, 10, 14 and 28 dpf; n = 8 per group. (F) Three-point bending test was performed on femurs at 6 weeks post-fracture; n = 6 per group. (G) Representative Safranin O/Fast green staining images of fracture calluses at 7, 10, 14 and 28 dpf; scale bar = 500 µm. (H) Callus area, cartilage area, and bone area were measured at 7, 10, 14 and 28 dpf; n = 6 per group. Data are presented as means ± SD. Statistical analysis was performed using two-tailed Student's t test (B, D, E, F, H). ns = not significant, *P < 0.05, **P < 0.01, ****P < 0.0001.

The data so far demonstrated that EGFR signaling in stem/progenitor cells plays an important role in normal bone formation. Next, to test whether Prx1^+^ cells also contribute to bone regeneration and to investigate the participation of EGFR signaling in bone fracture repair, we created a midshaft stabilized femoral fracture model in 2-month-old Prx1-Cre; Rosa-tdTomato mice to investigate the presence and distribution of Prx1^+^ and HBEGF^+^ cells during fracture. Consistent with our earlier observation in non-injured mice, the non-fractured femur exhibited prominent tdTomato expression in the articular cartilage, metaphysis and periosteum. In contrast, immunofluorescent staining showed that HBEGF was detectable in only a few Prx1^+^ cells within the periosteum and bone marrow (Figure 2A). However, 10 days post fracture (dpf), strong tdTomato expression was detected throughout the fracture callus, including both the bony and cartilaginous regions. Immunofluorescent staining for HBEGF indicated that the number and distribution of HBEGF-positive cells also dramatically increased within callus, and about 55% of HBEGF^+^ cells were also tdTomato^+^ (Figure 2A-B). Clearly, these data indicated that Prx1^+^ cells that expressed HBEGF contributed to both cartilage and bone formation within the callus in the fracture healing process.

Next, we sought to investigate the effect of HBEGF overexpression in Prx1^+^ cells during fracture repair. According to micro-CT analysis, callus formation (TV) in WT mice reached a peak at 10-14 dpf and then gradually decreased up to 28 dpf, but the fracture gap was still clearly visible. In contrast, *HBEGF Over^Prx1^* mice had much bigger calluses which reached a peak at 10 dpf and then almost disappeared by 28 dpf. Notably, the fracture line in *HBEGF Over^Prx1^* mice at 28 dpf was nearly invisible with significantly better modified radiographic union score for tibia fractures (mRUST) healing scores (Figure 2C-E). Moreover, BV/TV significantly increased in *HBEGF Over^Prx1^* mice at all time points compared to WT mice, suggesting increased mineralization in *HBEGF Over^Prx1^* mice during fracture repair. Biomechanical properties of the fractured femurs, evaluated by three-point bending test, showed a remarkable increase in peak load (81%), stiffness (15%) and energy to failure (43%) in *HBEGF Over^Prx1^* mice compared to WT mice (Figure 2F).

We next used histological methods to investigate the underlying cellular mechanism involved in this accelerated fracture repair. As shown by Safranin O/Fast green staining, WT mice showed normal fracture healing processes, with both callus and cartilage tissue reaching a peak at around 7-10 dpf, after which callus was resorbed and converted into bone at 14 dpf. By 28 dpf, massive woven bone could still be visualized around the fracture site (Figure 2G). At the same time point, *HBEGF Over^Prx1^* mice showed accelerated formation of cartilaginous callus from day 7 onwards and prominent absorption of cartilaginous callus and initiation of mineralization at 14 dpf, which were reflected by 25% and 22% increases of cartilage area at 7 and 10 dpf, and 36% and 14% increases of bone area at 10 and 14 dpf, respectively. In these mice, fracture repair was almost completed at 28 dpf, with lamellar bone present at the fracture site (Figure 2G-H). Finally, we did not observe any obvious morphologic changes in heart, liver, spleen, lung or kidney in WT and *HBEGF Over^Prx1^* mice (Figure S3A). Taken together, these results demonstrated that EGFR signaling plays an important role in bone fracture healing, and that activation of this signaling pathway significantly accelerates the fracture healing process without causing any side effects on any major internal organs.

### EGFR overactivation enhances the response of periosteal progenitors to fracture injury

**Figure 3:**
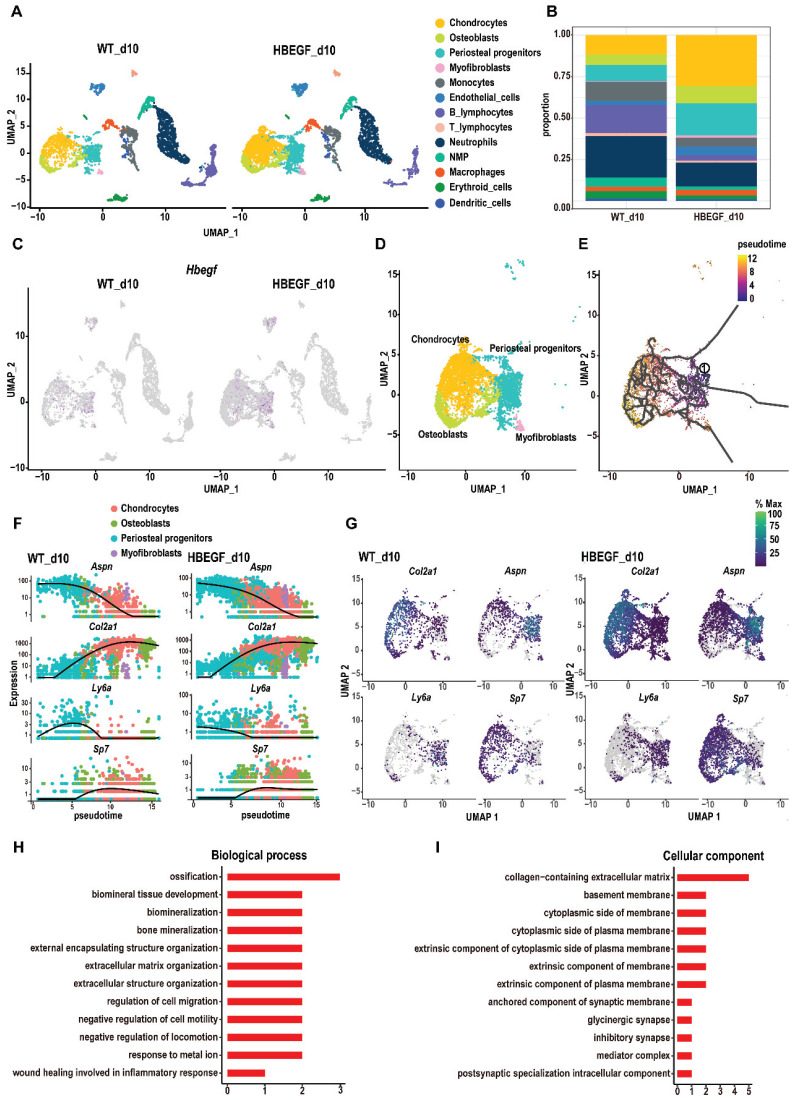
** Single-cell RNA sequencing of callus at 10 days post-fracture.** (A) The uniform manifold approximation and projection (UMAP) plot of callus isolated from WT and *HBEGF Over^Prx1^* mice by single-cell transcriptomics. (B) Stacked bars showing the percentage of each cell population within callus isolated from WT and *HBEGF Over^Prx1^* mice, based on the UMAP distribution. (C) UMAP plot of *HBEGF* expression pattern in WT and* HBEGF Over^Prx1^* mice. (D) UMAP visualization of non-hematopoietic cells (including periosteal progenitors, chondrocytes, osteoblasts and myofibroblasts) within callus isolated from WT and *HBEGF Over^Prx1^* mice. (E) Pseudotime trajectory analysis of periosteal progenitors, chondrocytes, osteoblasts and myofibroblasts. (F) Expression of fibrogenic (*Aspn*), chondrogenic (*Col2a1*), mesenchymal (*Ly6a*) and osteoblastic (*Sp7*) lineage marker genes over pseudotime in WT and *HBEGF Over^Prx1^* mice. (G) Feature plot of *Col2a1*, *Aspn*, *Ly6a* and *Sp7* expression, in WT and *HBEGF Over^Prx1^* mice. (H, I) Gene ontology (GO) term analysis of genes upregulated in the periosteal progenitor cluster in the *HBEGF Over^Prx1^* group compared to the WT group.

To dissect the underlying cellular mechanism involved in responding to EGFR during fracture healing, we isolated callus surrounding the fracture site from WT and *HBEGF Over^Prx1^* mice at 10 dpf for single-cell RNA sequencing (scRNA-seq). We identified periosteal progenitors expressing *Prx1*, *Pdgfra*, *Ly6a* and *Acta2*; chondrocytes expressing *Acan* and *Sox9*; osteoblasts expressing *Runx2* and *Ibsp*; myofibroblasts expressing *Myl9*; endothelial cells expressing *Cdh5* and *Pecam*, and immune cell clusters (including neutrophils, neutrophil-myeloid progenitors (NMP), monocytes, macrophages, dendritic cells, T cells and B cells) (Figure 3A, S4A and Table S1). Additionally, periosteal progenitors, chondrocytes and osteoblasts were found to be more abundant in *HBEGF Over^Prx1^* mice compared to WT mice (Figure 3B). Interestingly, *HBEGF* was highly expressed in these cells (Figure 3C and S4B). Therefore, we then focused our analysis on these non-hematopoietic cell clusters that form cartilage and bone in the callus (Figure 3D). We performed pseudo-time analyses of these cells and found that the directionality of cell differentiation and diversification started from periosteal progenitors, then advanced into chondrocytes and ended in osteoblasts (Figure 3E and S4C-D). *In silico* trajectory analysis showed that periosteal progenitors express stem cell genes (*Ly6a*) and start upregulating fibrogenic genes (*Aspn*) before chondrogenic genes (*Col2a*) and osteogenic genes (*Sp7*). Notably, in comparison with the WT group, chondrogenic genes were upregulated, followed by osteogenic genes, in the *HBEGF Over^Prx1^* group in response to fracture (Figure 3F-G). In summary, these results indicate that periosteal progenitors are the ancestor of chondrocytes, osteoblasts and myofibroblasts, contributing to soft and hard callus formation during fracture healing, and the response of periosteal progenitors to fracture in *HBEGF Over^Prx1^* mice was strongly enhanced compared to the WT group.

Given the apparent developmental centrality of periosteal progenitors, we sought to analyze the differentially-expressed genes (DEGs) in periosteal progenitor clusters between WT and *HBEGF Over^Prx1^* groups. Compared to the WT group, gene ontology (GO) analysis revealed that upregulated genes in the periosteal progenitor cluster in the *HBEGF Over^Prx1^* group were highly relevant to the regulation of ossification, biomineral tissue development, cell migration and bone mineralization (Figure 3H-I).

### EGFR overactivation stimulates survival, migration and differentiation of periosteal progenitors

**Figure 4:**
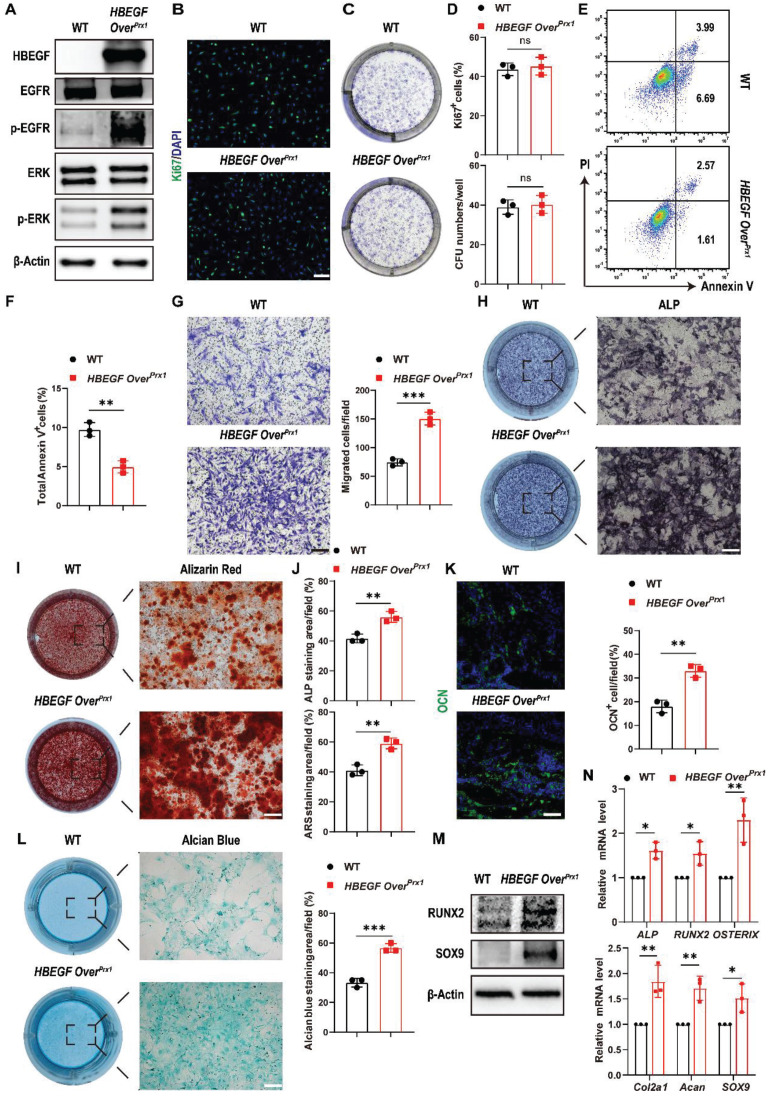
** HBEGF promotes survival, migration and differentiation of periosteal progenitors.** (A) Western blot of HBEGF and EGFR downstream signals in periosteal progenitors derived from WT and *HBEGF Over^Prx1^* mice. (B) Immunofluorescence staining of Ki67 in WT and *HBEGF Over^Prx1^* periosteal progenitors; scale bar = 200 µm. (C) CFU-F assay using periosteal progenitors dissociated from WT and *HBEGF Over^Prx1^* mice. (D) Percentages of Ki67^+^ cells or CFU^+^ cells were quantified; n = 3 per group. (Ki67, *P* = 0.6297; CFU, *P* = 0.7096). (E) Apoptosis was measured by flow cytometry. (F) Percentages of apoptotic cells were quantified; n = 3 per group. (G) Periosteal progenitors were seeded into the upper chamber in 1% FBS medium. Migrated cells on the lower surface of the membrane were stained with crystal violet, and the number of migrated cells was quantified; n = 3 per group. Scale bar = 100 µm. (H) ALP staining of WT and *HBEGF Over^Prx1^* periosteal progenitors after culture in osteogenic medium for 10 days. (I) Alizarin red S (ARS) staining of WT and *HBEGF Over^Prx1^* periosteal progenitors after culture in osteogenic medium for 21 days. (J) ALP- or ARS-positive areas were measured using Image J; n = 3 per group. (K) Representative images of OCN immunostaining (green) in fracture callus at 10 dpf, counterstained with DAPI (blue); scale bar = 100 µm. (L) Alcian blue staining of WT and *HBEGF Over^Prx1^* periosteal progenitors after culture in chondrogenic medium for 20 days. Alcian blue-positive areas were measured using Image J; n = 3 per group. (M) Western blot of RUNX2 and SOX9 in periosteal progenitors derived from WT and *HBEGF Over^Prx1^* mice. (N) RT-PCR analysis of osteogenic marker gene expression and chondrogenic marker gene expression in WT and *HBEGF Over^Prx1^* periosteal progenitors harvested after 2 weeks of culture in osteogenic or chondrogenic medium; n = 3 per group. Data are presented as means ± SD. Statistical analysis was performed using two-tailed Student's t test (D, F, G, J, K, L, N). ns = not significant, *P < 0.05, **P < 0.01, ***P < 0.001.

The spatiotemporal links between periosteal progenitors and HBEGF expression during the fracture healing process, described above, indicated that EGFR signaling may promote the differentiation of periosteal progenitors. To validate the above findings *in vitro*, we isolated periosteal progenitors from WT and *HBEGF Over^Prx1^* mice. Western blotting confirmed that there was increased HBEGF expression in periosteal progenitors of *HBEGF Over^Prx1^* mice, contributing to the activation of EGFR and ERK signaling, a major EGFR downstream signaling pathway, as shown by elevated levels of p-EGFR and p-ERK (Figure 4A). EGFR signaling is known to be essential for cell proliferation and survival 21. To our surprise, *HBEGF Over^Prx1^* periosteal progenitors showed no significant difference in the percentage of Ki67-positive or CFU^+^ cells (Figure 4B-D). CCK8 assay showed the same results (Figure S5A), suggesting that overactivation of EGFR signaling in periosteal progenitors has no apparent effect on their proliferative potential. However, HBEGF significantly protected cells against apoptosis compared to WT cells (Figure 4E-F). Furthermore, a transwell assay showed that HBEGF robustly enhanced the migration ability of periosteal progenitors (Figure 4G).

Next, we evaluated whether HBEGF regulates chondrogenic and osteogenic differentiation of periosteal progenitors. We found that HBEGF did stimulate osteogenic differentiation as shown by strong alkaline phosphatase (ALP) and Alizarin Red S (ARS) staining and higher expression of osteoblastic marker genes (*ALP*, *RUNX2* and *OSTERIX*) (Figure 4H, I, J, M and N). Immunofluorescence staining of OCN in the fracture callus at 10 dpf further confirmed our *in vitro* data (Figure 4K). Evaluation of chondrogenic differentiation by alcian blue staining and western blotting as well as RNA analysis of chondrogenic marker genes (*Col2a1*, *Acan* and *SOX9*) showed similar results (Figure 4L-N). Thus, HBEGF promoted survival, migration and differentiation of periosteal progenitors, thereby facilitating bone regeneration and repair.

### EGFR overactivation indirectly promotes vascularization during fracture repair

**Figure 5:**
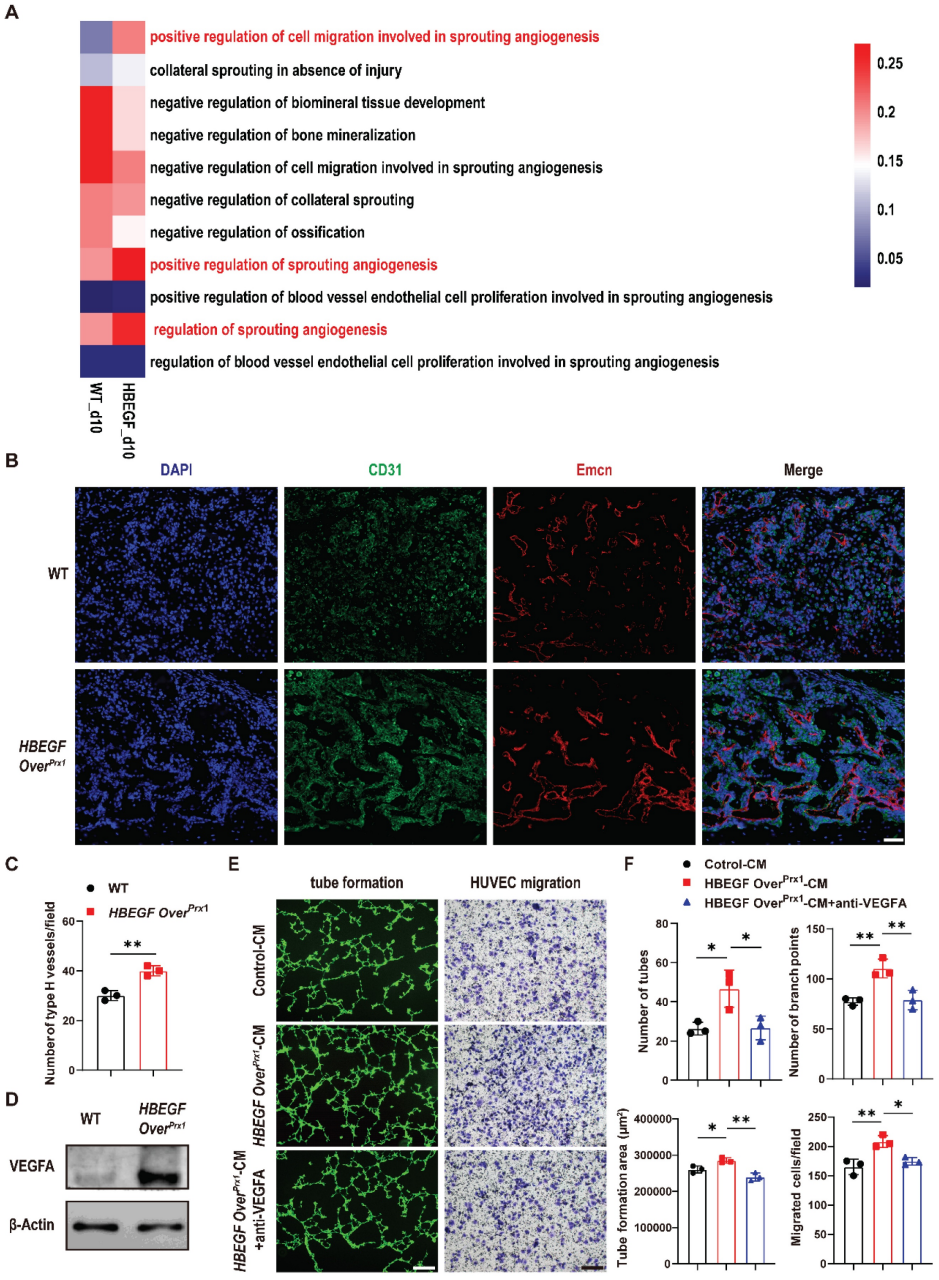
** Overexpression of HBEGF in stem/progenitor cells promotes angiogenesis in fracture callus.** (A) Gene set variation analysis heatmap showing the angiogenic gene sets in the periosteal progenitor cluster. (B) Representative co-immunostaining images of CD31 (green) and Endomucin (red) in fracture callus at 10 dpf, counterstained with DAPI (blue). Scale bar = 200 µm. (C) Quantification of the number of type H vessels within callus; n = 3 per group. (D) Western blot of VEGFA expression in periosteal progenitors derived from WT and *HBEGF Over^Prx1^* mice; n = 3 per group. (E) Representative images of capillary-like structures in HUVECs incubated with conditioned medium collected from WT periosteal progenitors (control-CM) or *HBEGF Over^Prx1^* periosteal progenitors (HBEGF Over^Prx1^-CM). For the migration assay, HUVECs were seeded into the upper chamber, then control-CM or HBEGF Over^Prx1^-CM was added to the lower chamber. Migrated cells on the lower surface of the membrane were stained with crystal violet. Scale bar = 100 µm. (F) Quantification of the tube formation number, branch points, tube formation area and migrated cell number; n = 3 per group. Data are presented as means ± SD. Statistical analysis was performed using two-tailed Student's t test (C, F). *P < 0.05, **P < 0.01.

Angiogenesis intimately coupled with osteogenesis facilitates successful progression of bone regeneration during fracture repair 31. By scRNA-seq, we found that the proportion of endothelial cells was increased in the *HBEGF Over^Prx1^* group (5.1%), compared to the WT group (2.4%) (Figure 3B). Coincidentally, *HBEGF* was also expressed in endothelial cells (Figure 3C). These findings led us to investigate whether HBEGF regulates angiogenesis during fracture repair. We first performed gene set variation analysis (GSVA) and single-sample gene set enrichment analysis (ssGSEA) of endothelial clusters but no obvious angiogenesis gene sets were upregulated in the *HBEGF Over^Prx1^* group compared to the WT group (Figure S6A-B). Interestingly, the expression of genes involved in sprouting angiogenesis in the *HBEGF Over^Prx1^* group was strikingly elevated in the periosteal progenitor cluster (Figure 5A, S6C and Table S2), suggesting that EGFR overactivation may promote angiogenesis in an indirect way. We next performed immunofluorescence staining of type H vessels (CD31^hi^Emcn^hi^) in fracture callus at 10 dpf and found abundant formation of type H vessels in *HBEGF Over^Prx1^* mice (Figure 5B-C). To validate our *in vivo* findings in *HBEGF Over^Prx1^* mice, we isolated periosteal progenitors and found the expression of vascular endothelial growth factor A (VEGFA) was markedly higher in *HBEGF Over^Prx1^* periosteal progenitors compared to WT (Figure 5D). Next, we collected conditioned medium (CM) from WT and *HBEGF Over^Prx1^* periosteal progenitors (referred to as control-CM and HBEGF Over^Prx1^-CM) for subsequent analysis in HUVECs. As shown in Figure 5E and F, tube formation and migration of HUVECs were both significantly enhanced when incubated with HBEGF Over^Prx1^-CM compared to those treated with control-CM, and these increases could be significantly blocked by VEGFA inhibition. However, both control-CM and HBEGF Over^Prx1^-CM resulted in no significant differences in proliferation or apoptotic changes in HUVECs (Figure S5B-C). Together, these results showed that overexpression of HBEGF in periosteal progenitors indirectly leads to angiogenesis at the fracture site, thus promoting fracture repair.

### Inhibition of EGFR signaling delays fracture healing and intramembranous cranial bone defect repair

**Figure 6:**
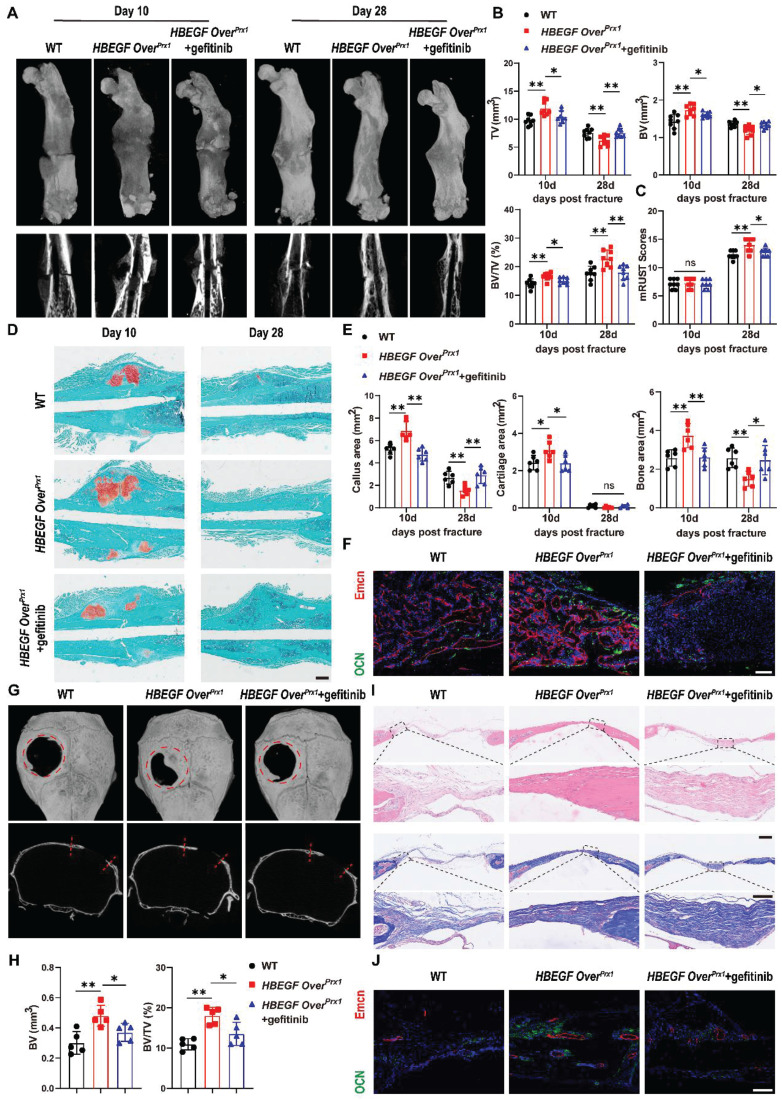
** Inhibition of EGFR signaling impairs fracture healing or intramembranous repair of calvarial defects.** (A) Representative 3D reconstructions and coronal cross-sectional micro-CT images of fracture callus at 10 and 28 dpf. (B) Tissue volume (TV), bone volume (BV) and bone volume fraction (BV/TV) of fracture callus at 10 and 28 dpf were analyzed; n = 8 per group. (C) Fracture healing scores were quantified based on mRUST Scoring Criteria at10 and 28 dpf; n = 8 per group. (D) Representative Safranin O/Fast green staining images of fracture calluses at 10 and 28 dpf. Scale bar = 500 µm. (E) Callus area, cartilage area and bone area were measured at 10 and 28 dpf; n = 6 per group. (F) Representative co-immunostaining images of OCN (green) and Endomucin (red) in fracture callus at 10 dpf, counterstained with DAPI (blue). Scale bar = 200 µm. (G) Representative 3D reconstructions (top) and sagittal cross-sectional (bottom) micro-CT images of bone defects at 6 weeks post-surgery. The boundaries of the original defect are indicated by the red dashed line. (H) Bone volume (BV) and bone volume fraction (BV/TV) of the bone defect area at 6 weeks post-surgery were analyzed; n = 5 per group. (I) Representative H&E and Masson's trichrome staining of calvarial bone defects at 6 weeks post-surgery. Magnified images of the boxed areas are shown in the panel below. Scale bar = 500 μm (upper image); 100 μm (lower image). (J) Representative co-immunostaining images of OCN (green) and Endomucin (red) in calvarial defects at 6 weeks post-surgery, counterstained with DAPI (blue). Scale bar = 100 µm. Data are presented as means ± SD. Statistical analysis was performed using one-way ANOVA with Bonferroni's post-hoc test for multiple comparisons (B, C, E, H). ns = not significant, *P < 0.05, **P < 0.01.

To further illustrate the participation and function of EGFR signaling in fracture healing, we treated *HBEGF Over^Prx1^* mice with the EGFR-specific inhibitor gefitinib once every other day after fracture 28. The levels of EGFR activation indicators, p-EGFR, p-ERK and HBEGF, were significantly decreased in the fracture callus of gefitinib-treated mice at 10 dpf (Figure S7). Interestingly, administration of gefitinib slightly but significantly blocked the effect of EGFR on bone mass at 3 months of age, with decreases in BV/TV, TB. N and Ct. TH (Figure S8A-B). More importantly, we observed that gefitinib-treated mice had much smaller callus, with significant reductions in TV, BV and BV/TV at 10 dpf. By 28 dpf in *HBEGF Over^Prx1^* mice, fractures were almost bridged, indicating an accelerated fracture healing process, but those in gefitinib-treated *HBEGF Over^Prx1^* mice were lagging, resulting in a significantly decreased mRUST healing score (Figure 6A-C). Safranin O/Fast green staining further confirmed a reduced callus size at 10 dpf and a delayed healing process, with callus still remaining at 28 dpf in gefitinib-treated mice (Figure 6D). Quantification of callus area, cartilage area and bone area showed that all were significantly reduced by 31%, 23% and 30%, respectively, at 10 dpf (Figure 6E). Consistent with the results of micro-CT and histology, administration of gefitinib resulted in fewer OCN^+^ cells and Endomucin^+^ blood vessels within fracture callus in *HBEGF Over^Prx1^* mice (Figure 6F). These data showed that HBEGF overexpression-induced accelerated endochondral fracture healing was completely abolished by gefitinib.

Our data so far have demonstrated that EGFR overactivation in skeletal progenitors plays a critical role in endochondral ossification. To further extend the above findings, we carried out another experiment using a critical-size (4 mm) calvarial defect mouse model which heals mainly through intramembranous ossification 32. Micro-CT showed that WT mice still had an obvious defect at 6 weeks after surgery, with slight bone formation observed near the edges of the defect. Importantly, *HBEGF Over^Prx1^* mice demonstrated substantial bone formation at 6 weeks that formed enough bony bridging to span most of the defect, but this effect was completely abolished by gefitinib treatment (Figure 6G). In line with these observations, micro-CT analysis confirmed the decrease in defect volume and increase in BV/TV in *HBEGF Over^Prx1^* mice compared with WT mice. Again, these healing effects of *HBEGF Over^Prx1^* were dramatically blocked by gefitinib (Figure 6H). We next performed histological analysis to investigate the regenerated tissues and found that in *HBEGF Over^Prx1^* mice, the defect area was filled with thin newly-formed bone, whereas only several layers of fibrous connective tissues and minimal new bone were observed in WT mice. The administration of gefitinib to *HBEGF Over^Prx1^* mice caused an appreciable reduction in bone formation in the defect area (Figure 6I). Further TRAP staining demonstrated the presence of reduced numbers of TRAP^+^ osteoclasts in *HBEGF Over^Prx1^* compared to WT mice (Figure S9A). In addition, we found stronger OCN^+^ cells and Endomucin^+^ blood vessels in *HBEGF Over^Prx1^* mice (Figure 6J). Once again, this robust osteogenesis coupled to the angiogenic effects of *HBEGF Over^Prx1^* were abolished by gefitinib. Taken together, our data suggest that EGFR activation by HBEGF promoted bone regeneration via both endochondral and intramembranous ossification.

### HBEGF promotes survival, migration and differentiation of periosteal progenitors and mediates angiogenesis via EGFR/ERK signaling

**Figure 7:**
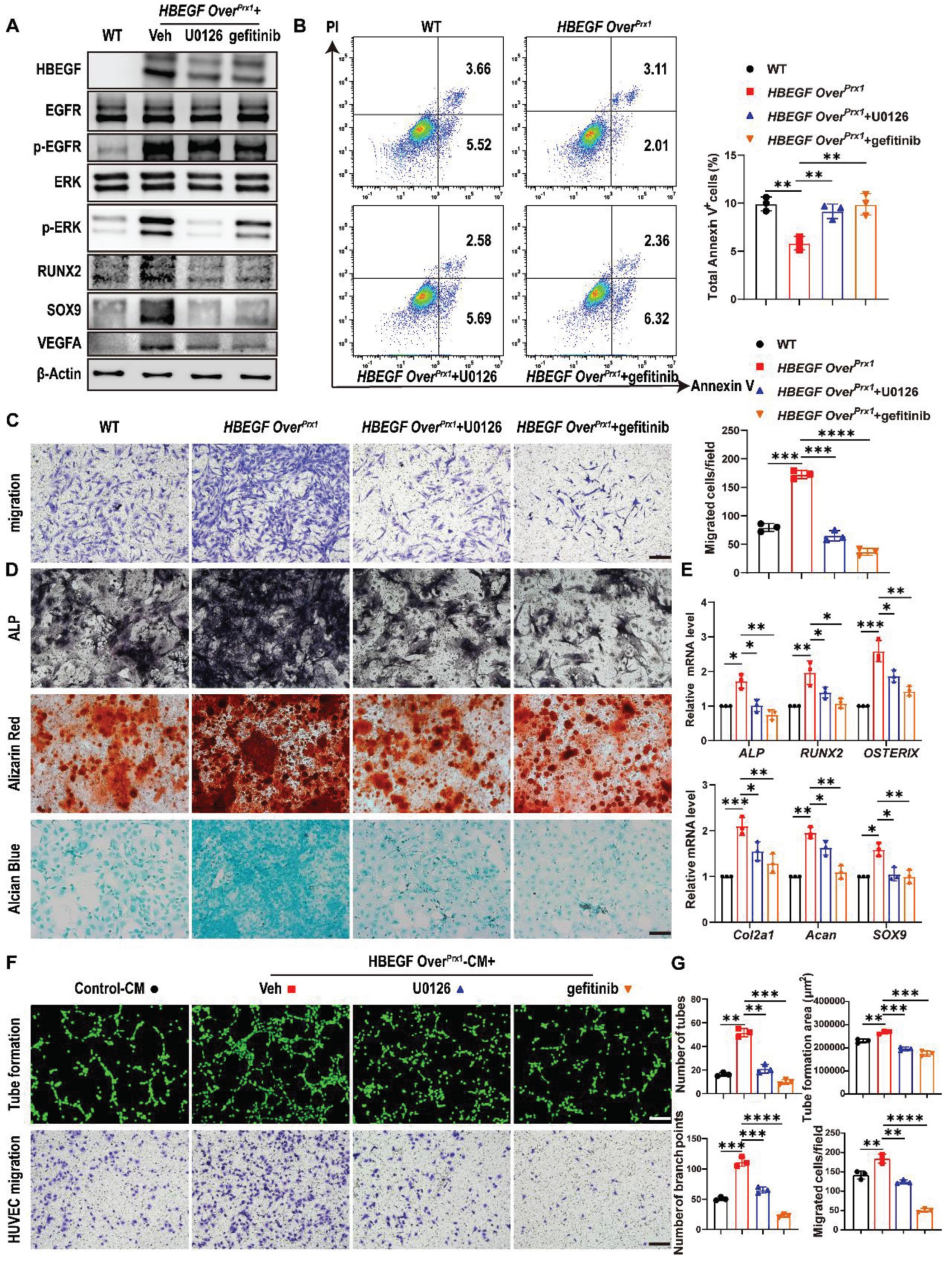
** HBEGF activates EGFR/ERK signaling to promote survival, migration and differentiation of periosteal progenitors.** (A) Western blot of HBEGF, RUNX2, SOX9, VEGFA and EGFR downstream signals in periosteal progenitors derived from WT and *HBEGF Over^Prx1^* mice. *HBEGF Over^Prx1^* periosteal progenitors were treated with or without gefitinib (10 μM) or U0126 (10 μM); n = 3 per group. (B) Apoptosis in the indicated periosteal progenitors, measured by flow cytometry. Percentages of apoptotic cells were quantified; n = 3 per group. (C) Periosteal progenitors were seeded into the upper chamber in 1% FBS medium. Migrated cells on the lower surface of the membrane were stained with crystal violet, and the number of migrated cells was quantified; n = 3 per group. Scale bar = 100 µm. (D) ALP, ARS and alcian blue staining of the indicated periosteal progenitors after culture in osteogenic or chondrogenic medium. (E) RT-PCR analysis of osteogenic marker gene expression and chondrogenic marker gene expression in the indicated periosteal progenitors harvested after 2 weeks of culture in osteogenic or chondrogenic medium. n = 3 per group. (F) Tube formation and HUVEC migration assays were performed with control or HBEGF Over^Prx1^ conditioned medium in the absence or presence of U0126 or gefitinib. Scale bar = 100 µm. (G) Quantifications of the tube formation number, branch points, tube formation area and migrated cell number; n = 3 per group. Data are presented as means ± SD. Statistical analysis was performed using one-way ANOVA with Bonferroni's post-hoc test for multiple comparisons (B, C, E, G). *P < 0.05, **P < 0.01, ***P < 0.001, ****P < 0.0001.

HBEGF binds and signals through the EGFR to activate the downstream ERK pathway 33. Therefore, to further determine whether the migration and differentiation of periosteal progenitors and their angiogenesis are mediated via EGFR/ERK signaling, gefitinib and U0126, a specific inhibitor targeting ERK, were used to block signal transduction in *HBEGF Over^Prx1^* periosteal progenitors. Western blotting demonstrated that both gefitinib and U0126 inhibited the expression of HBEGF, p-EGFR and p-ERK in *HBEGF Over^Prx1^* periosteal progenitors (Figure 7A). As expected, U0126 or gefitinib had no significant influence on cell proliferation (Figure S10A-B) but significantly abolished the inhibitory effect of HBEGF on apoptosis (Figure 7B). In addition, HBEGF-driven migration of periosteal progenitors was almost completely blocked by U0126 or gefitinib (Figure 7C). Next, we examined the role of U0126 and gefitinib in HBEGF-mediated osteogenic and chondrogenic differentiation of periosteal progenitors and found that HBEGF-induced differentiation and expression of osteoblastic and chondrogenic markers were both appreciably inhibited by U0126 or gefitinib (Figure 7A, D, E and S10C). To further clarify the regulatory mechanism of U0126 and gefitinib on angiogenesis, we treated *HBEGF Over^Prx1^* periosteal progenitors with U0126 or gefitinib and found that HBEGF-induced VEGFA expression was greatly inhibited by U0126 or gefitinib (Figure 7A). In accordance with these findings, HUVEC migration and tube formation were substantially enhanced when cultured with HBEGF Over^Prx1^-CM, however, these effects were markedly abolished by the administration of either U0126 or gefitinib (Figure 7F-G). Thus, our data strongly suggest that HBEGF signals through EGFR/ERK to upregulate various osteogenic, chondrogenic and angiogenic mediators, which contributes to fracture repair.

## Discussion

In the present study, we provide genetic evidence that HBEGF plays a critical role in bone remodeling and regeneration. We found that Prx1-HBEGF overexpressing mice showed an increase in bone mineralization as well as bone mass and substantially accelerated fracture healing. Using scRNA-seq analysis, we found that periosteal progenitors, chondrocytes and osteoblasts made up a large portion of the cell population and were rapidly expanded within callus after fracture. Periosteal progenitors lie at the center of a developmental path that gives rise to chondrocytes and osteoblasts.

Intriguingly, HBEGF was highly enriched in periosteal progenitors and their derived chondrocytes and osteoblasts, and was upregulated within callus after fracture, indicating a positive association between HBEGF and fracture repair. Further *in vivo* and *in vitro* findings suggested that HBEGF acted through EGFR/ERK signaling to stimulate the survival, migration and differentiation of periosteal progenitors, thus promoting chondrogenesis and osteogenesis as well as angiogenesis within the callus and ultimately contributing to fracture healing.

In this study, we observed an intriguing phenomenon: the femurs of *HBEGF Over^Prx1^* mice (13.84 ± 0.41 mm) were generally shorter than those of WT mice (14.77 ± 0.26 mm). Considering that bone lengthening is closely associated with the development of growth plates 34, and that Prx1-labeled cells were also abundant throughout the height of the growth plate (Figure 1A), we then compared the growth plate development pattern between WT and *HBEGF Over^Prx1^* mice at 1 and 3 months of age. The results revealed that at 1 month of age, the growth plate of *HBEGF Over^Prx1^* mice was modestly expanded (WT: 347.7 ± 23.09 μm; *HBEGF Over^Prx1^*: 430.8 ± 29.90 μm), but by 3 months of age, the growth plate exhibited more pronounced shrinkage compared to WT mice (WT: 241.0 ± 28.84 μm; *HBEGF Over^Prx1^*: 259.1 ± 39.29 μm). Based on these data, we reason that overexpression of EGFR signaling in Prx1-labeled cells may affect the development of the growth plate during postnatal life, thus leading to a shortened long bone phenotype. However, the precise mechanisms underlying this regulation require further investigation in future studies.

Several EGFR activation or deficient mouse models have been used to investigate the role of EGFR in bone metabolism. Mice with reduced EGFR activity in osteoblast lineage cells, generated using *EGFR^f/f^ Runx2-Cre* (*EGFR^ΔOb^*) mice, exhibit a low-bone-mass phenotype with less calcified bone and fewer bony trabeculae 35. *Col-Cre EGFR^Wa5/f^* mice also show a low-bone-mass phenotype due to decreased bone formation and increased bone resorption 36. Similar to our work, a previous study took advantage of the DTR to generate Dermo1-Cre HBEGF overexpressing (*HBEGF Over^Dermo1^*) mice. However, these mice showed decreases in bone mineral density, bone mass, and the number and thickness of trabeculae 37. In addition, BMSCs isolated from* HBEGF Over^Dermo1^* mice exhibited strong proliferative ability but limited osteogenic and chondrogenic potentials 37. Both Dermo1-Cre and Prx1-Cre mouse lines have been used to mark early skeletal progenitor cells but Prx1 may mark earlier osteogenic progenitors than Dermo1 38. Dermo1 is a basic helix-loop-helix transcription factor that is highly expressed in mesodermal tissues during embryogenesis 39. During skeletal development, Dermo1 is expressed at high levels in condensed mesenchyme that will give rise to cartilage and bone and later in the perichondrial and periosteal tissues that give rise to osteoblasts 40. A 2.4 kb Prx1 promoter directs the transgene expression in osteochondro progenitor cells in the developing limb buds as early as embryonic day 10.5 (E10.5) 41, and its expression is later extinguished in the condensing mesenchyme and chondrocytes but persists in the perichondrium/periosteum at E15.5 12. Consequently, considering Dermo1-Cre broadly targets mesenchymal lineage cells 40, while Prx1-Cre is more specifically expressed in stem/progenitor cells 42, this could explain the differences. A direct comparison between the different models is also complicated by the fact that different mouse strains and different ubiquitous promoters were employed 43. Therefore, we suggest that the therapeutic efficacy of EGFR ligands depends on their activity and specificity. In addition, osteoblastic marker genes were increased in *HBEGF Over^Prx1^* mice, a finding which contradicts some *in vitro* experiments in which adding exogenous EGFR ligands to osteoblastic cells inhibited bone marker genes such as *ALP*, type 1 collagen, *BSP*, *OCN* and the osteoblastic-specific transcription factor *RUNX2* 44, 45. We reason that the dosage of EGFR ligands used *in vitro* and the *in vivo* versus *in vitro* conditions might contribute to this discrepancy. However, further mechanistic studies are needed in the future to address this discrepancy between these *in vitro* and *in vivo* data.

To date, there have been contradictory reports regarding the function of EGFR in osteoclasts. Some *in vivo* and *in vitro* data show that EGFR signaling directly increases the number of mature osteoclasts in mouse bone marrow cultures 46, 47. In addition to the direct effects of EGFR signaling on osteoclasts, several studies have also indicated that in rheumatoid arthritis (RA), EGFR ligands, such as EGF and TGF-α, induce RA synovial fibroblasts to produce various pro-inflammatory cytokines such as tumor necrosis factor-alpha (TNF-α) and interleukin-6 (IL-6), thereby further activating osteoclast precursor cells (monocytes/macrophages), promoting osteoclastogenesis, and ultimately leading to bone erosion 48, 49. In contrast, another study by Zhang *et al.* showed that activating osteoblastic EGFR activity actually decreased osteoclast formation and bone resorption 24. However, Markus *et al.* generated *LysM-Cre EGFR^f/f^* mice (*EGFR^ΔOc^*) and found that they did not display any bone defects nor any differences in the number of osteoclasts in trabecular bones or in serum CTX 35. Consistent with Zhang *et al.*, our work here found that *HBEGF Over^Prx1^* mice showed no significant change in serum CTX, but a marked decrease in the number of TRAP^+^ osteoclasts was observed in trabecular bones, suggesting that EGFR signaling may not directly regulate osteoclastogenesis. Indeed, exogenous EGFR ligands show little effect on osteoclast differentiation 37, but can serve as paracrine and juxtacrine factors to regulate RANKL, OPG or M-CSF expression in osteoblasts, which in turn affect osteoclastogenesis.

Fracture healing is a precisely regulated multistage process involving multiple cell lineages. Periosteum is a tissue that responds rapidly to injury, makes a major cellular contribution to both cartilage and bone, and promotes angiogenesis during fracture healing 50. Lack of periosteum or damage to periosteal progenitors has been reported as the predominant reason for delayed fracture healing or fracture non-union 51, 52. Given the important role of periosteum during fracture healing, extensive studies have previously been performed using one or a combination of markers to identify periosteal progenitors by flow cytometry or lineage tracing approaches. Our study sought to shift the current research pattern that has mainly focused on the identification of periosteal progenitor cell markers, and instead focus on investigating the function of a specific cell type (Prx1^+^ periosteal progenitor) before and after fracture. Using a comprehensive approach that combined cell lineage tracing, scRNA-seq and *in vitro* studies, we determined that the cells labeled by Prx1 are periosteal progenitors that contribute to both chondrocyte and osteoblast production within the fracture callus. These cells secrete various cytokines and chemokines in response to callus formation after fracture, which has been shown to be a periosteal response 53. Notably, HBEGF was highly expressed in periosteal progenitors and their derived cells within callus. Immunofluorescent staining also revealed that the number of Prx1-positive and HBEGF-positive cells were both dramatically increased after fracture, and about 55% of Prx1^+^ periosteal progenitors expressed HBEGF. These data indicate that periosteal progenitors are the main responding cells of the EGFR pathway during fracture healing. Additionally, HBEGF expression and function in Prx1^+^ periosteal progenitors appear to be crucial for effective fracture healing, as demonstrated by enhanced chondrogenesis and osteogenesis within callus through endochondral ossification. While most bones are formed through endochondral ossification, the clavicles and the cranial bones are formed by intramembranous ossification, during which mesenchymal cells differentiate directly into osteoblasts without the involvement of chondrocytes 54. Here, we also observed enhanced bone formation in a calvarial defect mouse model in *HBEGF Over^Prx1^* mice, indicating that EGFR activation by HBEGF in Prx1^+^ periosteal progenitors could promote bone regeneration via both endochondral and intramembranous ossification. In addition to the regenerative potential, periosteal progenitors also produce secretory mediators that promote angiogenesis and modulate the immune response 55, 56. Indeed, an adequate blood supply is critical for delivering various growth factors and cytokines to the fracture site, leading to successful fracture repair 57. In this study, our data showed that overexpression of HBEGF in periosteal progenitors resulted in increased VEGFA expression, thereby indirectly promoting HUVEC migration and tube formation. These *in vitro* observations were further reinforced by our *in vivo* data showing enhanced type H vessel formation within callus in *HBEGF Over^Prx1^* mice. Overall, EGFR activation by HBEGF promoted chondrogenesis and osteogenesis of periosteal progenitors, and also enhanced angiogenesis through a possible paracrine effect of periosteal progenitors. These effects together contributed to cartilage callus formation and turnover and mineralized bone formation, thus boosting fracture healing.

In summary, our study is the first to raise the important notion that HBEGF is an overall regulator and stimulator of the periosteal progenitor-mediated fracture healing process. The ability of HBEGF to play multiple biological roles makes it ideal as a therapeutic protein for bone repair and regeneration. However, a nonnegligible concern is the possible carcinogenic effect of activating EGFR signaling. High EGFR activity due to missense mutations, deletions, and insertions is frequently observed in tumors including those in the breast, lung, liver, and brain 58. Therefore, we overactivated EGFR activity only in stem/progenitor cells to minimize this possibility. No obvious morphologic changes were observed in vital internal organs in *HBEGF Over^Prx1^* mice up to 3 months of age. However, long-term pharmacological trials should still be performed before translating our findings to the clinic in future. Nevertheless, the physiological function and the apparent promotion of fracture repair resulting from EGFR activation that we demonstrate here provide a reasonable basis and premise for investigating the clinical application of exogenous EGFR ligands. And this represents just the first step in translating our research findings into clinical applications, because in future studies, we will explore nanocarrier-based drug delivery systems to deliver exogenous EGFR ligands to periosteal progenitors at the fracture site, thereby significantly enhancing the therapeutic efficacy of EGFR ligands in bone repair. Furthermore, the structure of the conjugates, the potential use of different EGFR ligands and their dosage should be optimized to achieve favorable therapeutic outcomes. Such comprehensive studies will provide critical insights for developing exogenous EGFR ligand-based therapies to treat inadequate bone repair and regeneration disorders.

## Materials and Methods

### Animals

Prx1-Cre mice were crossed with Rosa-DTR mice to generate *Prx1-Cre DTR* (*HBEGF Over^Prx1^*) mice and their WT (DTR-or Cre-only) siblings. Prx1-Cre mice were crossed with ROSA-tdTomato mice to generate *Prx1-tdTomato* mice. All mouse strains were purchased from the Jackson laboratory and these mice were kept at the specific-pathogen-free (SPF) facility under standard animal care and feeding conditions (12 h light, 12 h dark cycle, with free access to food and water). Mouse experiments performed in this study were approved by the Animal Care and Ethics Committee at Huazhong University of Science and Technology (Ethical approval number: 4111).

### OVX, femoral fracture and calvarial defect models

Bilateral OVX was performed to induce osteoporosis in 2-month-old female mice under anesthesia, the sham control mice underwent the same procedure but without removal of the ovaries 59. After 6 weeks, the mice were sacrificed, and samples were collected for subsequent experiments.

Femoral fractures were created in 2-month-old mice as previously described 52. Briefly, the right femur was exposed after anesthesia, then a sterilized 23-gauge needle was inserted into the medullary cavity, temporarily withdrawn to facilitate transection of the femur with scissors at the midshaft, and then reinserted to fix the fractures. Fractured femurs were harvested at 7, 10, 14 and 28 dpf for micro-computed tomography (μCT) and histology.

A critical-size calvarial defect model was prepared as previously described 60. Briefly, 2-month-old male mice were anesthetized, and a 4-mm diameter bone defect was created in the right parietal bone using a sterile punch, taking extreme care not to disturb the underlying dura mater. The defect was rinsed with sterile normal saline to remove any debris. Mice were sacrificed at 6 weeks after surgery and the calvariae were harvested for subsequent experiments.

For EGFR inhibitor treatment, mice were treated with gefitinib (100 mg/kg; LC Laboratories) via oral gavage once every other day for 4 or 6 weeks after surgery.

### Micro-CT analysis

Mouse specimens were harvested, and fixed in 4% paraformaldehyde (PFA) for 48 h, then scanned using a SkyScan 1176 high-resolution microscopic CT imaging system (μCT) at 9 μm resolution, with a 1 mm aluminum filter, 90 kV voltage, and 273 μA current. Volume reconstruction, three-dimensional image generation and analysis of related parameters, including total volume (TV), bone volume (BV), and BV/TV were recorded and evaluated using CTAN 1.12 software (Bruker MicroCT, Kontich, Belgium). Fracture healing progress was assessed based on mRUST scoring with use of micro-CT scanning 61.

### Mechanical testing

The fractured femurs were harvested at 6 weeks post fracture for a three-point bending test (span length, 10 mm; loading speed 1.8 mm/min) using an Instron 5542 (Instron, Norwood, MA, USA) as described previously 52. Peak load, stiffness, and energy to failure were calculated from the force-to-failure curve.

### Histology

Following micro-CT scanning, specimens were collected and fixed with 4% PFA overnight, decalcified at room temperature with 10% EDTA for 21 days and then embedded in paraffin. A series of 6 μm sections were subjected to staining with Safranin-O/Fast green, hematoxylin and eosin (H&E), Masson's trichrome and TRAP. Cartilage area, bone area and osteoclast surface per bone surface (Oc.S/BS) were quantified on Safranin-O/Fast green, TRAP-stained sections using Image J software ((NIH, Bethesda, MD, USA).

Paraffin sections were used for immunohistochemistry. After appropriate antigen retrieval, the sections were incubated with primary antibodies against EGFR (1:100, CST, Danvers, MA, USA; 4267), p-EGFR (1:200, Abcam, Cambridge, MA, USA; ab40815), ERK (1:200, CST, 4695), p-ERK (1:100, CST, 4370), HBEGF (1:100, Boster, Wuhan, China; A01759-3) and OCN (1:100, Boster, PB1008), followed by incubating with the appropriate secondary antibodies and color development using DAB (Vector Laboratories Ltd., Peterborough, UK).

### Immunofluorescence

For immunostaining, bone sections were blocked with 5% bovine serum for 30 minutes, then incubated with primary antibodies, including anti-HBEGF (1:100, Boster, A01759-3), anti-OCN (1:100, Boster, PB1008), anti-CD31 (1:50, 563607, BD Biosciences, Franklin Lakes, NJ, USA) and anti-Endomucin (1:100, sc-65495, Santa Cruz Biotechnology Santa Cruz, CA, USA) overnight at 4°C, washed twice with PBS, then incubated with secondary antibody for 1 hour at 37 °C, and washed with PBS before counterstaining with 4',6-diamidino-2-phenylindole (DAPI; Sigma-Aldrich, St Louis, MO, USA) and imaging under a confocal microscope (Nikon A1; Nikon, Tokyo, Japan).

### Single-cell RNA sequencing analyses

Callus was isolated and dissociated into a single-cell suspension by enzyme digestion from WT and *HBEGF Over^Prx1^* mice at 10 dpf (n = 5) as previously described 62. Briefly, the single-cell suspension was converted to a barcoded scRNA-seq library using the scRNA-seq library kit v3 (10 × Genomics, Pleasanton, CA, USA) according to the manufacturer's protocol. Libraries were sequenced using an Illumina NovaSeq 6,000 (Illumina Inc., San Diego, CA, USA) with read length of 150 bp by the Wuhan Biobank Co., Ltd. The R package Seurat (Version 3.0.2) was used for analysis of scRNA-seq data 63. Briefly, single cells expressing > 200 genes including < 20% of mitochondrial genes were retained for analysis; genes expressed in < 10 cells were not taken into account. Clustering was performed using the first 20 principal components with 0.5 as resolution and were visualized using the Uniform Manifold Approximation and Projection (UMAP). Integrated analysis was performed using the top 2,000 features and the 20 first principal components with a resolution set at 0.5. Differentially-expressed genes (DEGs) were defined by a *P* value threshold < 0.05 and log FC > 0.25. For Gene Ontology (GO) analyses, DEGs were used to find enriched functions using Enrich R software (https://amp.pharm.mssm.edu/Enrichr/) 64. GO functions including < 5 genes and with adjusted *P* value > 0.05 were excluded. Pseudotime and RNA velocity analysis were performed using Monocle3 v0.2.3.0 as previously reported 65.

### Enzyme-Linked Immunosorbent Assay (ELISA)

To analyze serum PINP, OCN, TRAP and CTX levels in mice, blood samples of WT and *HBEGF Over^Prx1^* mice were collected using serum separator tubes. The blood samples were centrifuged for 20 minutes at 3,000 × g at 4°C and the supernatant was harvested and stored at -20°C until measurement. The concentrations of PINP, OCN, TRAP and CTX in serum were measured using ELISA kits according to the manufacturer's protocol (both from Bio-Swamp, Wuhan, China. PINP: MU30602; CTX: MU30091; OCN: MU30420; TRAP: MU33117).

### Bone periosteal progenitor cell culture and treatment

Bone periosteal progenitor cells were harvested from 8-week-old mice as described previously 52. Briefly, intact femurs and tibias were isolated free of adherent soft tissue and both ends were carefully removed at the growth plate sites. The remaining bone fragments were digested in 0.2% collagenase A and 0.25% trypsin at 37 °C in an orbital shaker for 1 h. The cells in the supernatant were collected and seeded in growth medium (α-MEM containing 15% fetal bovine serum, 55 μM β-mercaptoethanol, 2 mM glutamine, 100 IU/mL penicillin, and 100 μg/mL streptomycin). All experiments were performed using passage 1 (P1) periosteal progenitor cells.

To test the effects of EGFR inhibitor (gefitinib; HY-50895, Med Chem Express, China) and ERK inhibitor (U0126; HY-12031A, Med Chem Express, China) on migration and differentiation, the cells were pretreated with the indicated concentrations of inhibitors (10 μM) for 6 h and treatment then continued throughout the whole assay 35.

For proliferation and apoptosis assays, periosteal progenitor cells were cultured on slides. After fixation with 4% PFA, cells were stained with a primary antibody against Ki67 (1:200, Thermo Fisher Scientific, Waltham, MA, USA; 14-569895) overnight at 4 °C. Next day, the cells were incubated with secondary antibody and then counterstained with DAPI to visualize the nuclei. Images were obtained under a confocal microscope (Nikon A1).

For CFU-F assay, 1 × 10^3^ cells were seeded into a 6-well plate and cultured for 10 days followed by crystal violet staining. The number of CFU-F was counted under a microscope.

Apoptosis was assessed using an Annexin V-FITC/PI Apoptosis Detection Kit (Vazyme, A211-02) according to the manufacturer's instructions.

For osteogenic differentiation, cells were cultured at 3.0 × 10^4^/well in a 12-well plate in osteogenic medium (α-MEM containing 10% FBS, 10 nM dexamethasone, 50 μg/mL ascorbic acid, 10 mM β-glycerophosphate, 100 IU/mL penicillin and 100 μg/mL streptomycin) for 2 weeks, followed by ALP or ARS staining.

For chondrogenic differentiation, confluent cells were cultured at 2 × 10^4^ cells/mL in chondrogenic medium (high-glucose DMEM, 100 μg/mL sodium pyruvate, 1% ITS + Premix, 50 μg/mL ascorbate-2-phosphate, 40 μg/mL L-proline, 0.1 mM dexamethasone, 10 ng/mL TGF-β1, 100 IU/mL penicillin, and 100 μg/mL streptomycin) for 3 weeks and then stained with alcian blue.

### Conditioned medium

Periosteal progenitor cells were seeded into a 6-well plate and cultured until they reached 80%-90% confluence, then washed three times with PBS and switched to serum free α-MEM. After 24 h, the conditioned medium was collected for migration and tube-formation assays using human umbilical vein endothelial cells (HUVECs).

### Migration assay

Cells (1 × 10^4^ cells/well) were seeded into the upper chamber of a 24-well Transwell™ plate containing serum-free medium. Then conditioned medium containing 15% FBS was added into the lower chamber. After incubating for 24 h, cells were fixed with 4% PFA and stained with crystal violet. The number of migrated cells was observed and quantified under a microscope.

### Tube formation

Vascular matrix gel was added to a 96-well plate on ice at a volume of 50 μL per well and allowed to solidify at 37 °C for 1 h. Then 2 × 10^4^ HUVECs were seeded into each well and incubated at 37 °C with 5% CO_2_ for 4 h. Cells were then stained with phalloidin (CA1640, Solarbio, Beijing, China) and photographed under a fluorescence microscope. The intersections and number of tubes formed were analyzed using Image J software.

### Real-time polymerase chain reaction (RT-PCR)

RNA was harvested from cells or callus tissues using Trizol reagent (TaKaRa Bio, Tokyo, Japan). A reversetranscription kit (TaKaRa Bio) was used to reverse transcribe mRNA into cDNA. After this, quantitative real-time PCR (qRT-PCR) was performed using a Power SYBR Green PCR Master Mix kit (Applied Biosystems, Foster City, CA, USA). The primer sequences for the genes used in this study are provided in Table S3.

### Western blot analysis

Total protein was extracted from cells or callus tissues using RIPA buffer (Thermo Fisher Scientific) supplemented with 1% phosphatase and proteinase inhibitors on ice, then quantified using the BCA assay (Sigma-Aldrich, St Louis, MO, USA). Next, proteins were subjected to sodium dodecyl sulfate polyacrylamide gel electrophoresis (SDS-PAGE) and transferred onto a 0.45 μm polyvinylidene difluoride membrane. The protein bands were detected using specific primary antibodies against EGFR (1:1000; CST, 4267), p-EGFR (1:1000; Abcam, ab40815), ERK (1:1000; CST, 4695), p-ERK (1:1000; CST, 4370), HBEGF (1:1000; Boster, A01759-3), β-Actin (1:4000; CST, 4970) and secondary antibodies. Signals on the membrane were detected using electrochemiluminescence (ECL, Pierce, Rockford, IL, USA).

### Statistical analysis

Statistical analyses and mapping were performed using GraphPad Prism 8 software (GraphPad Software Inc., LaJolla, CA, USA). Data are expressed as means ± SD. Comparisons between two groups were performed using two-tailed Student's t test. For comparison among three or more groups, data were analyzed by one-way analysis of variance (ANOVA) with the Bonferroni's post-hoc test. Values of *P* < 0.05 were considered statistically significant.

## Supplementary Material

Supplementary figures and tables.

## Abbreviations

EGFR: epidermal growth factor receptor
HBEGF: heparin-binding EGF-like growth factor
Prx1: paired-related homeobox gene-1
DTR: diphtheria toxin receptor
PINP: pro-collagen type I Nterminal propeptide
CTX: C-terminal telopeptide of type I collagen
OCN: osteocalcin
TRAP: tartrate-resistant acid phosphatase
dpf: days post fracture
TV: total volume
μCT: micro-computed tomography
BV: bone volume
CCK-8: cell counting kit-8
DAPI: 4',6-diamidino-2-phenylindole
scRNA-seq: single cell RNA sequencing
UMAP: uniform manifold approximation and projection
DEGs: differentially expressed genes
GO: gene ontology
HUVECs: human umbilical vein endothelial cells
KEGG: Kyoto encyclopedia of genes and genomes
GSVA: gene set variation analysis
ssGSEA: single-sample gene set enrichment analysis
RT-PCR: real-time polymerase chain reaction
VEGFA: vascular endothelial growth factor A
IHC: immunohistochemistry
FBS: fetal bovine serum
