# Supplementary material for: Overactivation of EGFR signaling in skeletal stem/progenitor cells promotes bone formation and repair
Source: Theranostics. 2025 Jul 24;15(16):8117–36. doi: 10.7150/thno.115406 (PMC12374589; doi:10.7150/thno.115406)
Supplement: Supplementary file 1 — Supplementary figures and tables. [file thnov15p8117s1.pdf]

Supplementary Materials for

Overactivation of EGFR signaling in skeletal stem/progenitor cells promotes bone formation and repair

Yuxiang Hu, Yangyang Chen, Xiaoyao Peng, Haitao Li, Guosilang Zuo, Hao Xu, Fashuai Wu, Yi Wang✉, Zengwu Shao ✉ ,Yulong Wei ✉

✉, Corresponding authors. Email: yulongwei@hust.edu.cn (Y.W.); szwpro@163.com (Z.S.); yi_wang@whu.edu.cn (Y. W.);

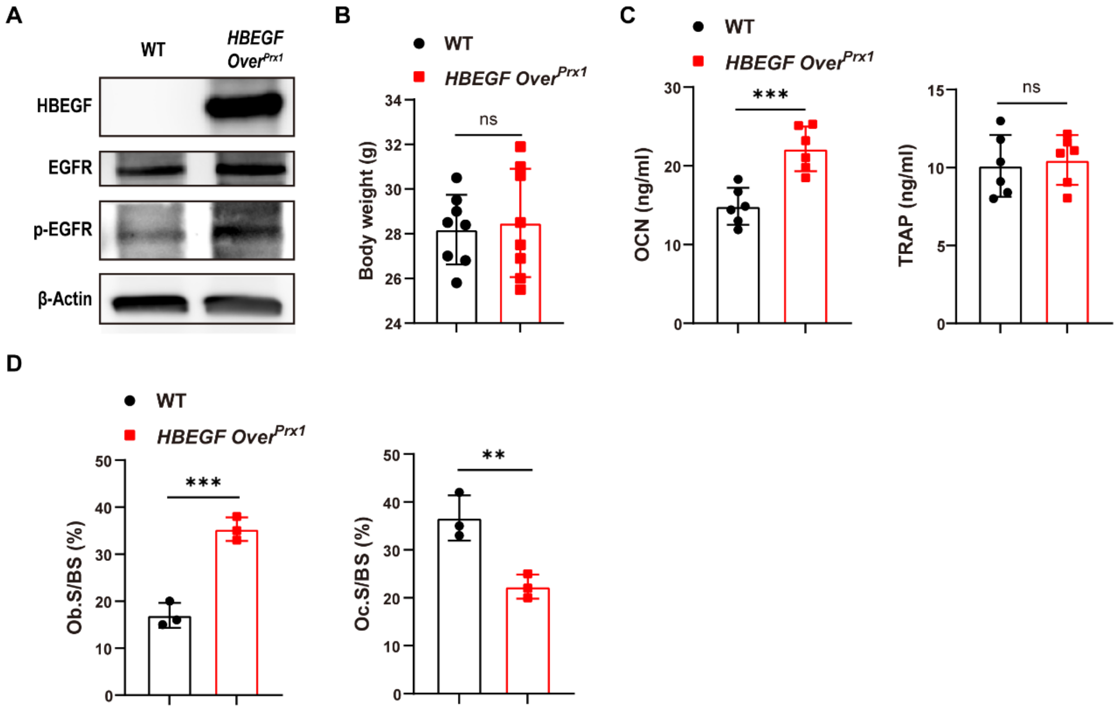

Figure S1. Mice that overexpress HBEGF in bone have normal body weight but stronger osteogenic ability. (A) Western blot of HBEGF and EGFR signals in bone tissues isolated from WT and *HBEGF Over*^{Prx1} mice. (B) The body weight of male WT and *HBEGF Over*^{Prx1} mice at 3 months of age; n = 8 per group. (C) Serum OCN and TRAP levels in WT and *HBEGF Over*^{Prx1} mice analyzed by ELISA; n = 6 per group. (TRAP: $P = 0.7272$). (D) Quantification of osteoblast surface (Ob.S)/bone surface (BS) and osteoclast surface (Oc.S)/bone surface (BS) from the WT and *HBEGF Over*^{Prx1} mouse femurs. Data are presented as means ± SD. Statistical analysis was performed using two-tailed Student's t test (B, C, D). ns = not significant. $^{**}P < 0.01$, $^{***}P < 0.001$.

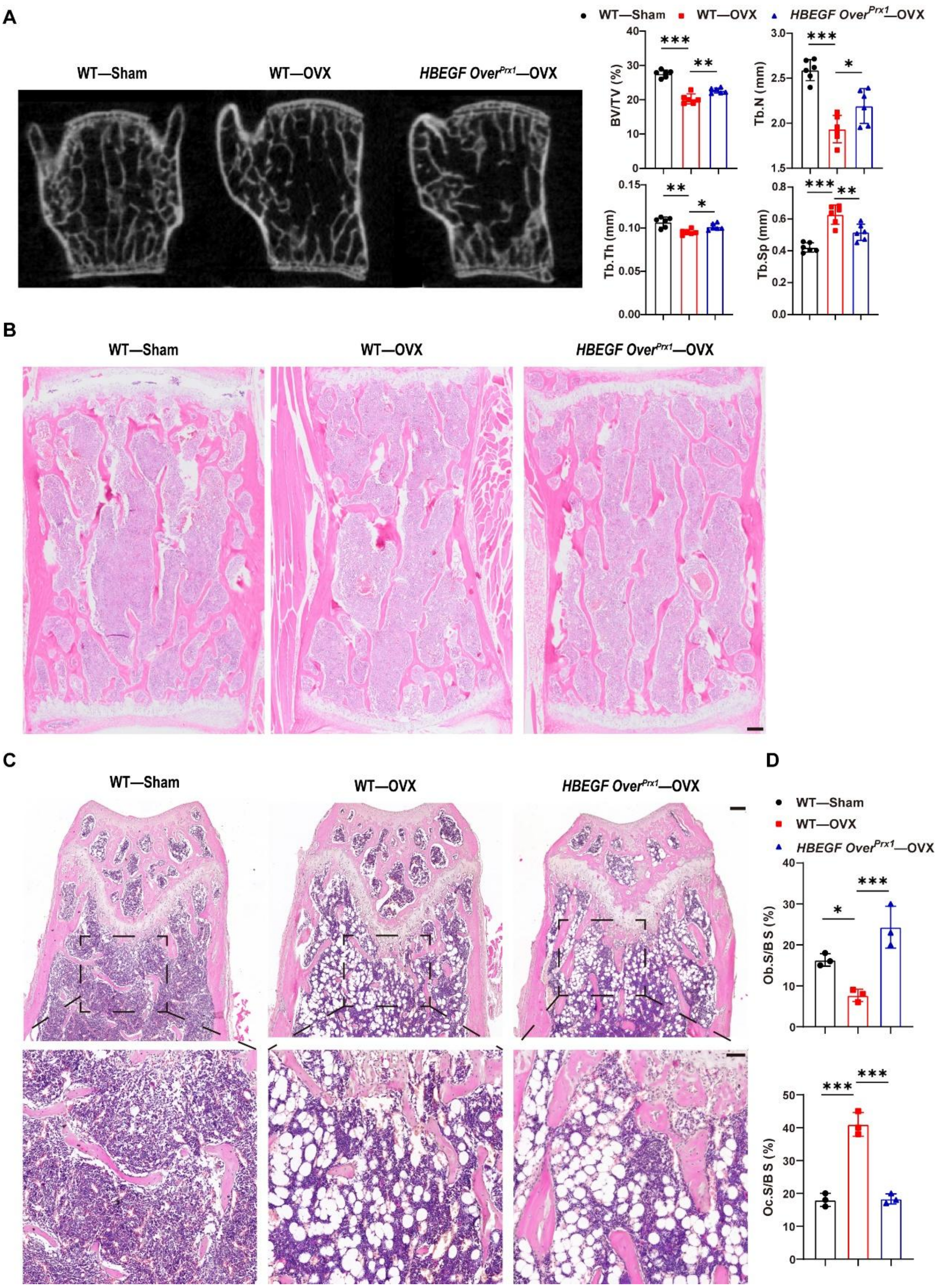

Figure S2. Activation of EGFR signaling rescues bone loss in an OVX mouse model.

(A) Representative 2D reconstructed micro-CT images of L5 vertebrae from 3-month-old WT and *HBEGF Over*^{Prx1} OVX mice. Quantification of the bone parameters including trabecular bone volume fraction (BV/TV), trabecular number (Tb. N),

trabecular thickness (Tb.TH) and trabecular separation (Tb. SP) based on micro-CT; n = 6 per group. (B) Representative H&E-stained images of L5 vertebrae at 4 weeks post-OVX surgery from WT and *HBEGF* $Over^{Prx1}$ mice. Scale bar = 500 μm. (C) Representative H&E staining images of distal femurs at 4 weeks post-OVX surgery from WT and *HBEGF* $Over^{Prx1}$ mice. Magnified images of the boxed areas are shown in the panel below. Scale bar = 500 μm (upper image); 100 μm (lower image). (D) Quantification of osteoblast surface (Ob.S)/bone surface (BS) and osteoclast surface (Oc.S)/bone surface (BS) from the indicated mouse femurs. Data are presented as means ± SD. Statistical analysis was performed using one-way ANOVA with Bonferroni's post-hoc test for multiple comparisons (A, D). $*P < 0.05$, $**P < 0.01$, $***P < 0.001$.

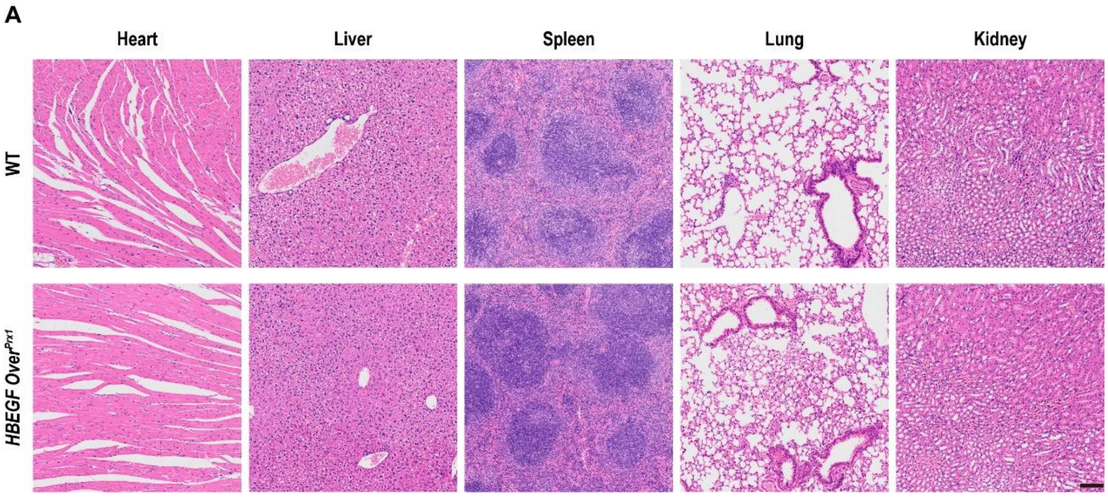

Figure S3. Overexpressing HBEGF in stem/progenitor cells does not affect vital internal organs. (A) H&E staining of representative organ sections from 3-month-old WT and *HBEGF* $Over^{Prx1}$ mice. Scale bars = 100 μm.

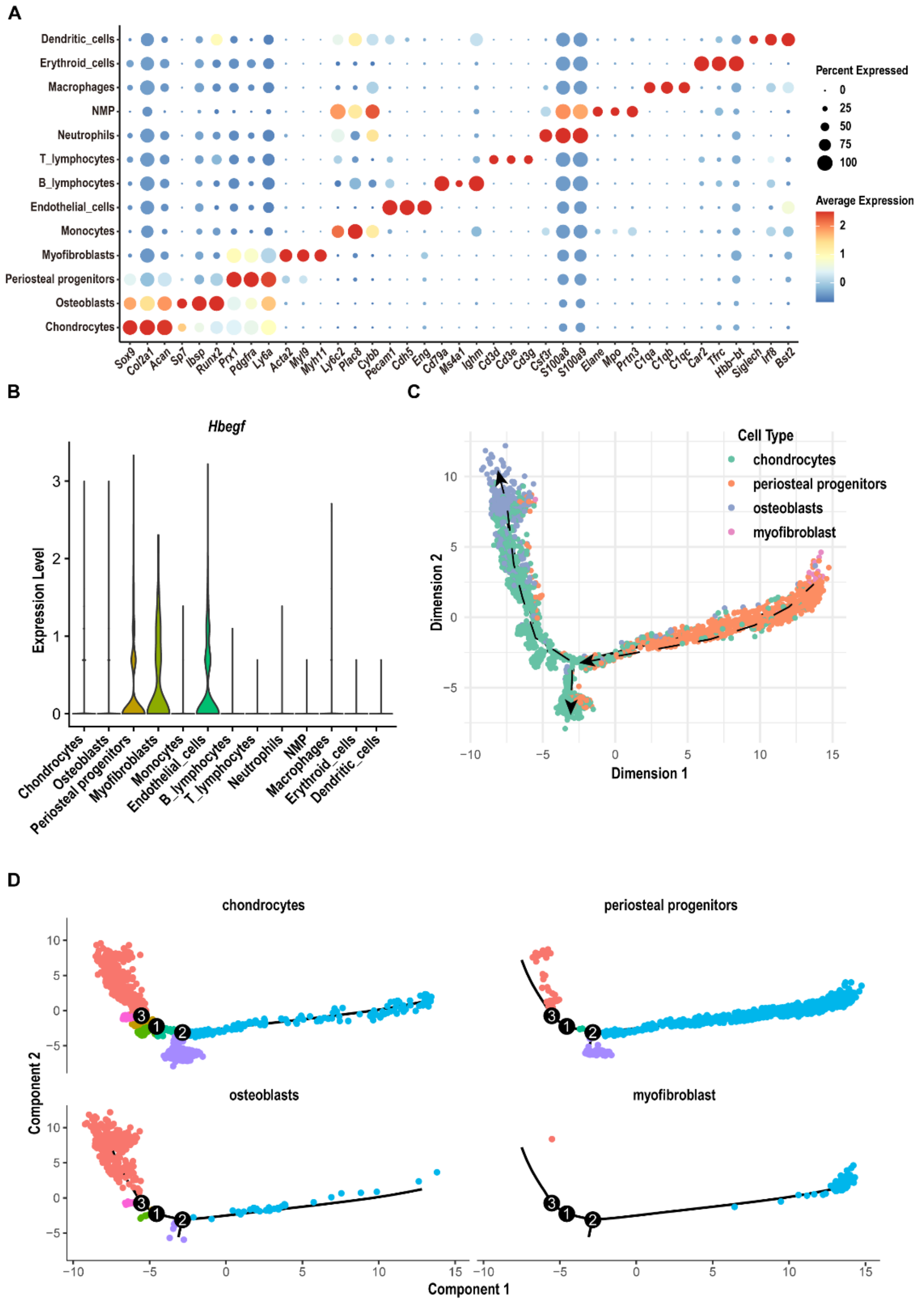

Figure S4. Expression pattern of *HBEGF* in fracture callus. (A) Expression of marker genes for cell types depicted by dot-plot. (B) Violin plot showing the expression distribution of the *HBEGF* gene based on scRNA-seq data. (C and D) Monocle pseudo-time trajectory showing the differentiation routes of periosteal progenitors during fracture healing.

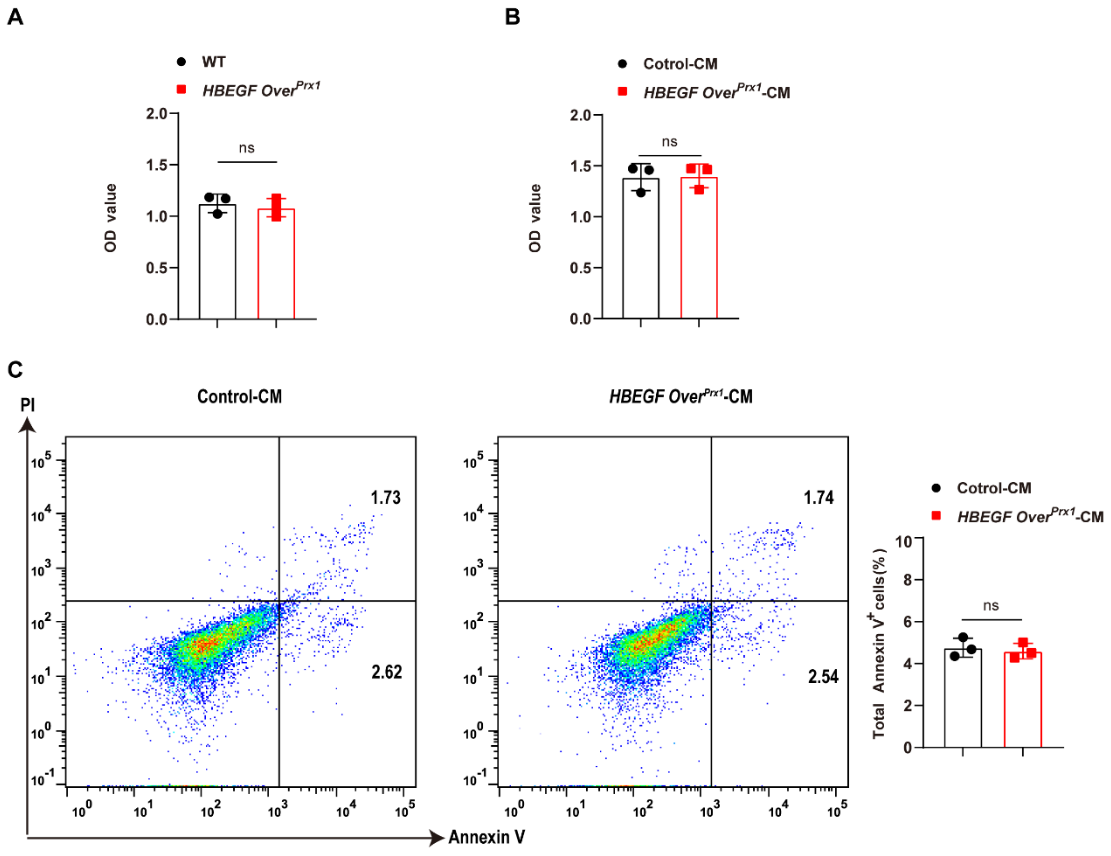

Figure S5. Overexpressing HBEGF in stem/progenitor cells does not affect the proliferation of periosteal progenitors or HUVECs. (A, B) Cell proliferation of periosteal progenitors (A) and HUVECs (B) was measured using CCK-8 assay; n = 3 per group (periosteal progenitors, P = 0.5945; HUVECs, P = 0.9163). (C) HUVEC apoptosis was measured by flow cytometry. Percentages of apoptotic cells were quantified; n = 3 per group (P = 0.6481). Data are presented as means ± SD. Statistical analysis was performed using two-tailed Student's t test (A, B, C). ns = not significant.

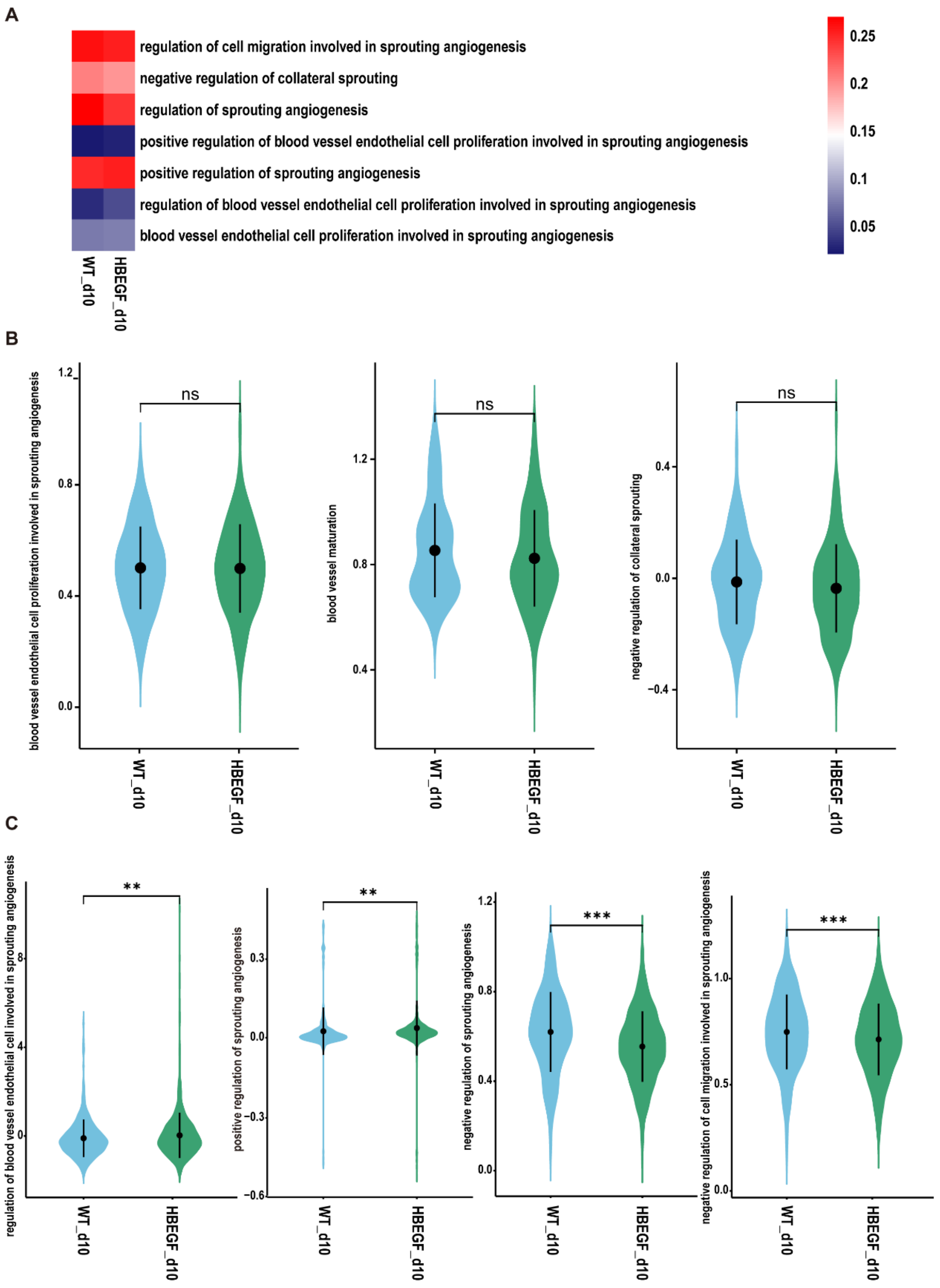

Figure S6. EGFR signaling activation in stem/progenitor cells promotes angiogenesis in fracture callus. (A) Gene set variation analysis heatmap showing the angiogenesis gene sets in the endothelial cluster. (B) Single-sample gene set enrichment

analysis (ssGSEA) of the endothelial cluster, based on scRNA-seq. (C) Single-sample gene set enrichment analysis (ssGSEA) of the periosteal progenitor cluster, based on scRNA-seq. ns = not significant, **$P < 0.01$, ***$P < 0.001$.

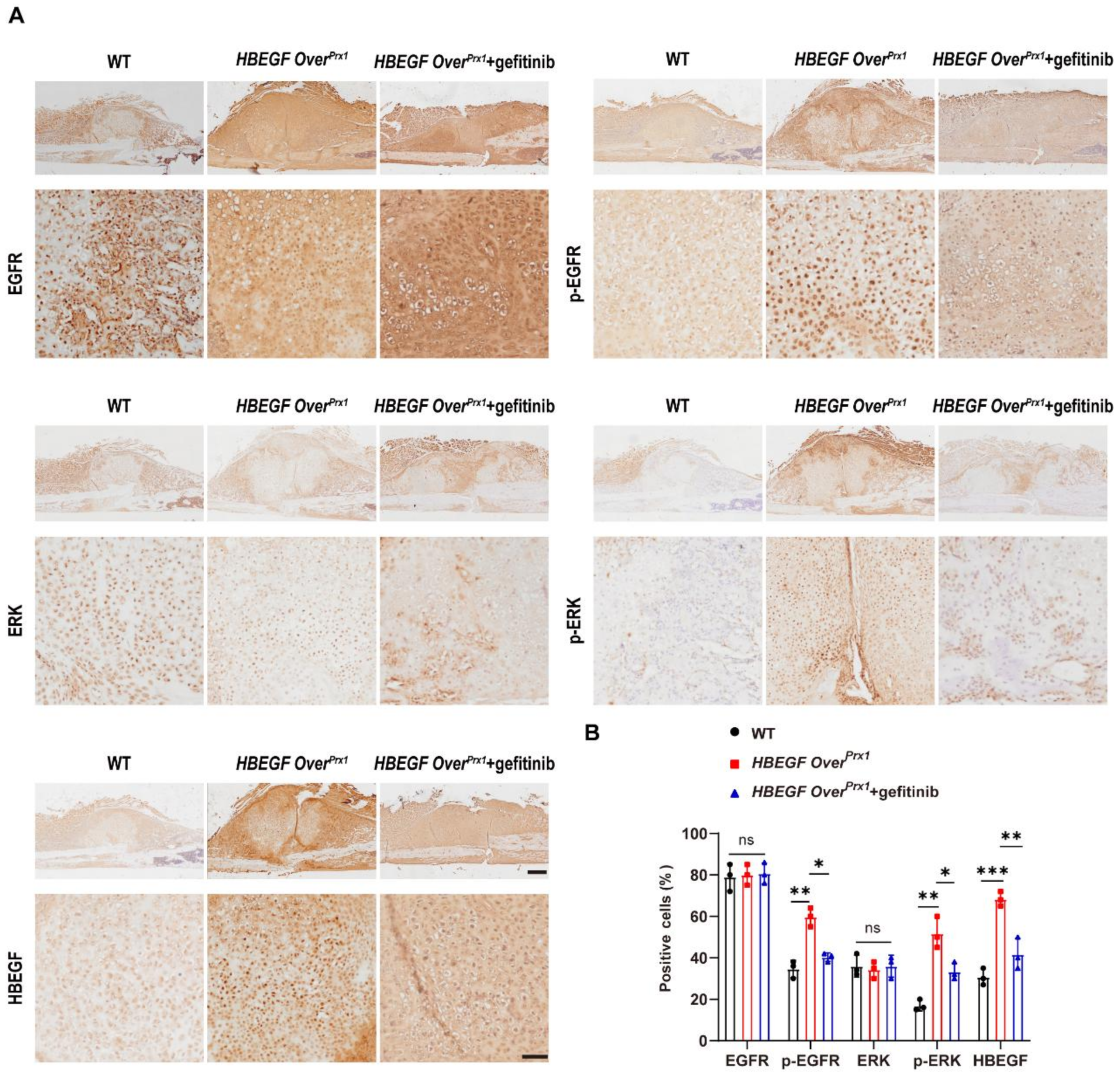

Figure S7. *HBEGF Over^{Prx1}* mice have increased HBEGF expression and EGFR activity in fracture callus, which is completely abolished by gefitinib. (A) Immunostaining of HBEGF, p-EGFR, EGFR, p-ERK and ERK within the callus of WT and *HBEGF Over^{Prx1}* mice. Scale bar = 500 μm (upper image); 100 μm (lower image). (B) The percentages of HBEGF^{+}, p-EGFR^{+}, EGFR^{+}, p-ERK^{+} and ERK^{+} cells within

callus were quantified; n = 3 per group. Data are presented as means ± SD. Statistical analysis was performed using one-way ANOVA with Bonferroni's post-hoc test for multiple comparisons (B). ns = not significant, *$P < 0.05$, **$P < 0.01$, ***$P < 0.001$.

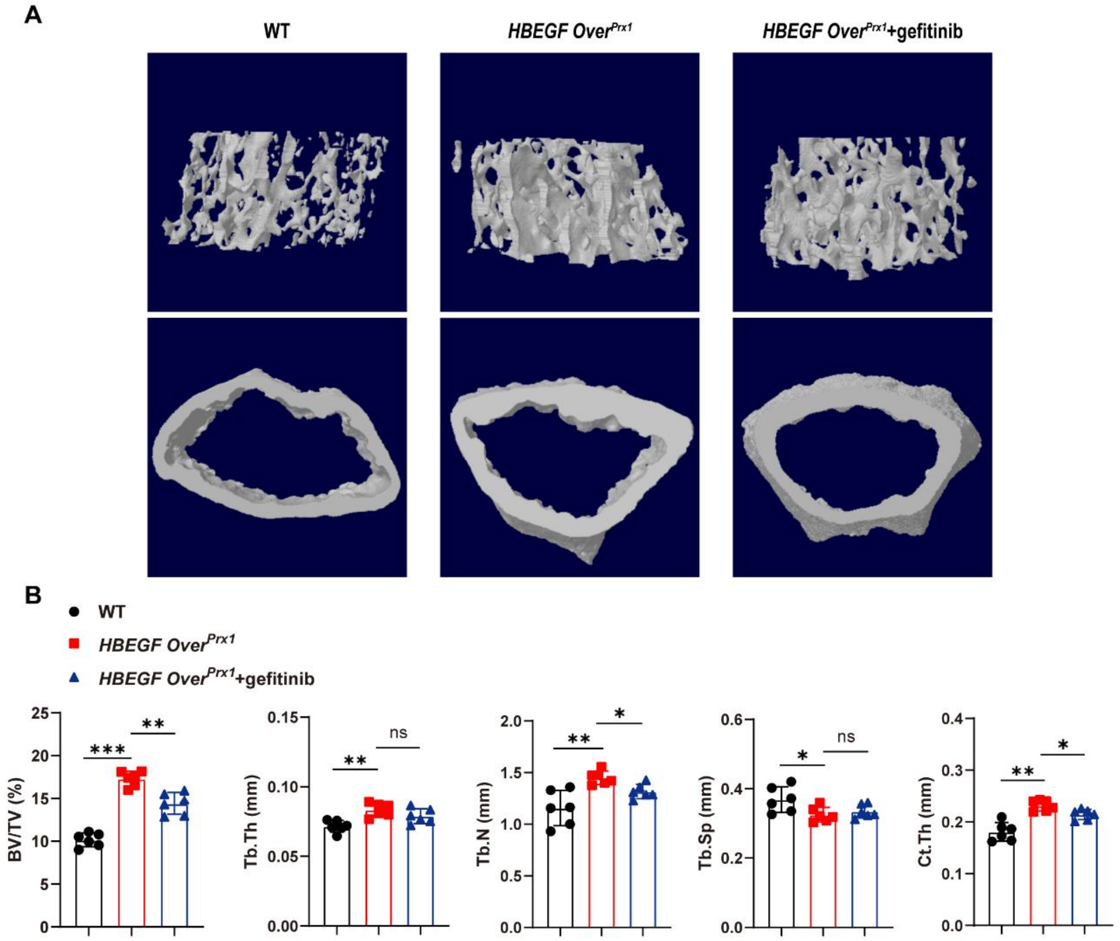

Figure S8. Gefitinib inhibits the high-bone-mass phenotype induced by overexpressing HBEGF in stem/progenitor cells. (A) Representative 3D reconstructed micro-CT images of trabecular architecture and cortical bone in the indicated mice at 3 months of age. (B) Quantification of the bone parameters including trabecular bone volume fraction (BV/TV), trabecular number (TB. N), trabecular thickness (TB.TH) and trabecular separation (TB. SP) based on micro-CT; n = 6 per group. Data are presented as means ± SD. Statistical analysis was performed using one-

way ANOVA with Bonferroni's post-hoc test for multiple comparisons (B). ns = not significant, *$P < 0.05$, **$P < 0.01$, ***$P < 0.001$.

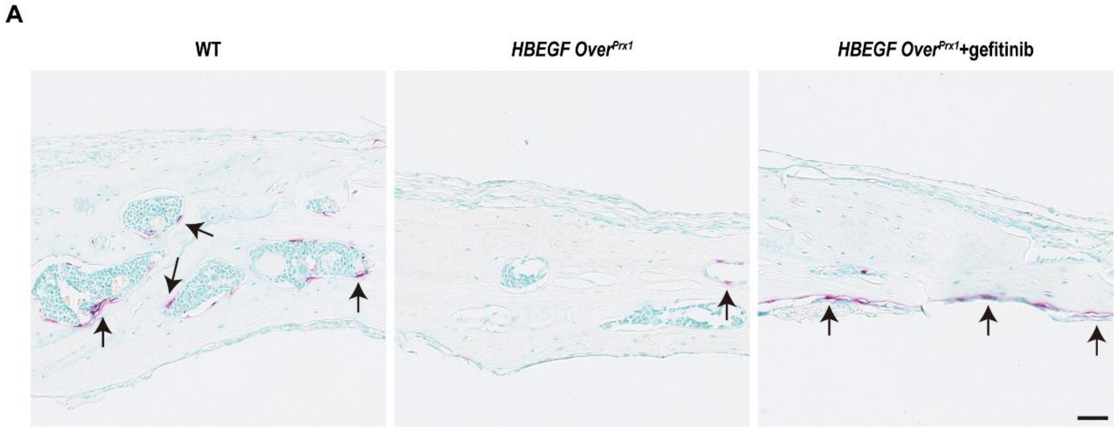

Figure S9. Gefitinib abolishes the protective effect of osteoclastogenesis in *HBEGF Over^{Prx1}* mice. (A) TRAP staining in calvarial bone defects at 6 weeks post-surgery. TRAP-positive cells in calvarial defects are indicated by black arrows. Scale bar = 100 μm.

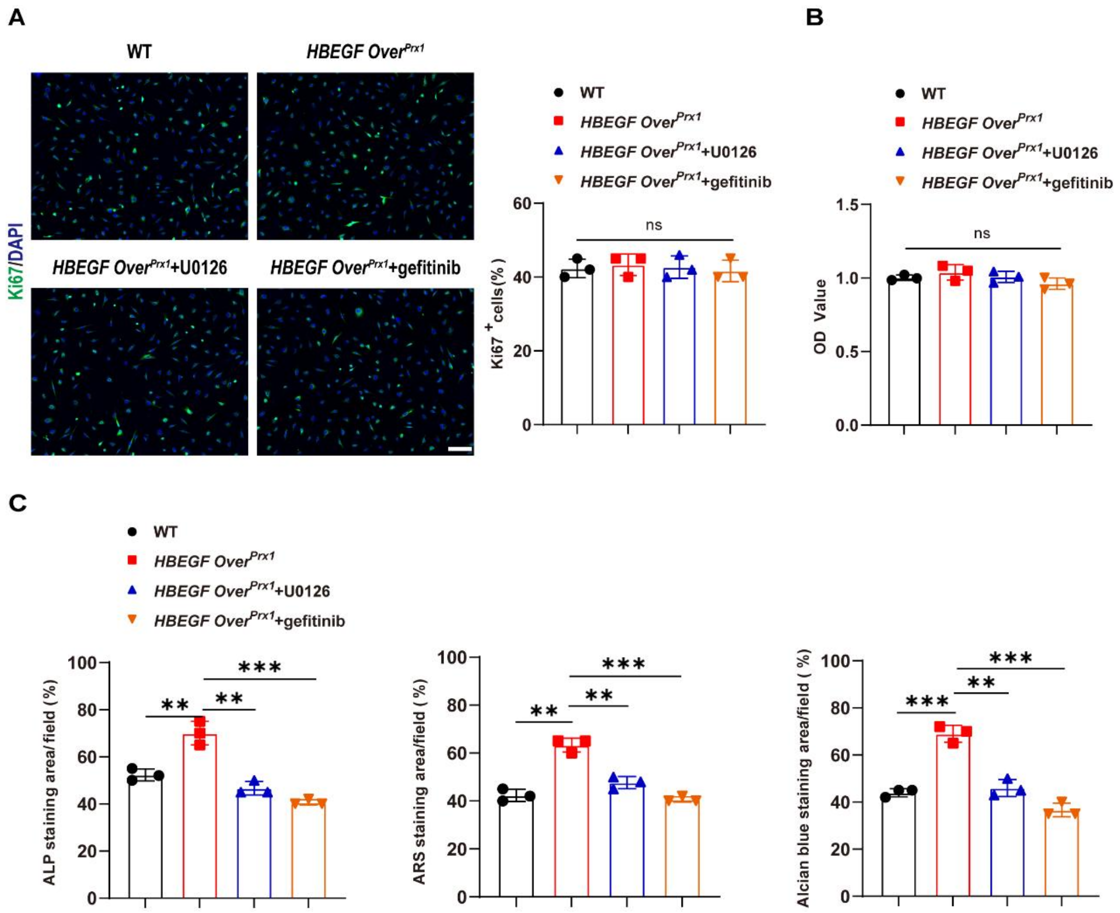

Figure S10. U0126 or gefitinib has no effect on proliferation but markedly inhibits differentiation of periosteal progenitors induced by HBEGF. (A) Immunofluorescence staining of Ki67 in the indicated periosteal progenitors. Scale bar = 200 μm. Percentages of Ki67^{+} cells were quantified; n = 3 per group. (B) Cell proliferation of periosteal progenitors was measured using CCK-8 assay; n = 3 per group. (C) ALP-, ARS- and Alcian blue-positive areas were measured using Image J; n = 3 per group. Data are presented as means ± SD. Statistical analysis was performed using one-way ANOVA with Bonferroni's post-hoc test for multiple comparisons (A, B, C). ns = not significant, $**P < 0.01$, $***P < 0.001$.

Table S1. Marker genes for cell populations used in this study.

Cell type	Cell marker
periosteal progenitors	*Prx1, Pdgfra, Ly6a, Acta2*
chondrocytes	*Acan, Sox9, Col2a1*
osteoblasts	*Runx2, Ibsp, Sp7*
myofibroblasts	*Myl9, Myh11*
endothelial cells	*Cdh5, Pecam1*
neutrophils	*Csf3r, S100a8, S100a9*
neutrophil-myeloid progenitors (NMPs)	*Elane, Mpo, Prtn3*
monocytes	*Ly6c2, Plac8, Cybb*
dendritic cells	*Bst2, lrf8, Siglech*
T cells	*Cd3d, Cd3e, Cd3g*
B cells	*Cd79a, Ms4a1, lhgm*
macrophages	*C1qa, C1qb, C1qc*
erythroid cells	*Car2, Tfrc, Hbb-bt*

Table S2. Gene sets involved in sprouting angiogenesis in the periosteal progenitor cluster.

Gene sets	WT	HBEGF
GOBP_POSITIVE_REGULATION_OF_CELL_MIGRATION_INVOLVED_IN_SPROUTING_ANGIOGENESIS	0.071740286	0.218458423
GOBP_COLLATERAL_SPROUTING_IN_ABSENCE_OF_INJURY	0.10725496	0.135489847
GOBP_NEGATIVE_REGULATION_OF_BIOMINERAL_TISSUE_DEVELOPMENT	0.255625016	0.163186441
GOBP_NEGATIVE_REGULATION_OF_BONE_MINERALIZATION	0.253804915	0.162228556
GOBP_NEGATIVE_REGULATION_OF_CELL_MIGRATION_INVOLVED_IN_SPROUTING_ANGIOGENESIS	0.258966849	0.203975856
GOBP_NEGATIVE_REGULATION_OF_COLLATERAL_SPROUTING	0.203726435	0.194647848
GOBP_NEGATIVE_REGULATION_OF_OSSIFICATION	0.204050522	0.150225964
GOBP_POSITIVE_REGULATION_OF_SPROUTING_ANGIOGENESIS	0.182705618	0.268448608
GOBP_POSITIVE_REGULATION_OF_BLOOD_VESSEL_ENDOTHELIAL_CELL_PROLIFERATION_INVOLVED_IN_SPROUTING_ANGIOGENESIS	0.019274016	0.024327586
GOBP_REGULATION_OF_SPROUTING_ANGIOGENESIS	0.188598776	0.251716553
GOBP_REGULATION_OF_BLOOD_VESSEL_ENDOTHELIAL_CELL_PROLIFERATION_INVOLVED_IN_SPROUTING_ANGIOGENESIS	0.031548158	0.036870399

Table S3. Real-time PCR primer sequences used in this study.

Gene	Forward primer (5' to 3')	Reverse primer (3' to 5')
Alp	ATCTTTGGTCTGGCTCCCATG	TTTCCCGTTCACCGTCCAC
Runx2	ACGAAAAATTAACGCCAGTCG	TCGGTCTGACGACGCTAAAG
Osterix	AGAGGTTCACTCGCTCTGACGA	TTGCTCAAGTGGTCGCTTCTG
Col2α1	GCTCCCAGAACATCACCTACCA	TCATTGGAGCCCTGGATG
Acan	CCTTGTCACCATAGCAACCT	CTACAGAACAGCGCCATCA
Sox9	AAGGGCTACGACTGGACG	ATGCGGGTACTGGTCTGC
Gapdh	GGTTGTCTCCTGCGACTTCA	TGGTCCAGGGTTTCTTACTCC
